# Socio-demographic disparities in health-related quality of life in hypertensive patients in Bangladesh: a comprehensive survey analysis

**DOI:** 10.1007/s11136-025-03912-3

**Published:** 2025-02-06

**Authors:** Md. Mizanur Rahman, Md. Nesar Uddin Sorkar, Ryota Nakamura, Md. Monirul Islam, Md. Ashraful Alam, Syed Khurram Azmat, Motohiro Sato

**Affiliations:** 1 Hitotsubashi Institute for Advanced Study, Hitotsubashi University, Tokyo, Japan; 2 Global Public Health Research Foundation, Dhaka, Bangladesh; 3 Graduate School of Economics, Hitotsubashi University, Tokyo, Japan; 4 Centre for Policy Studies, Cork University Business School, University College Cork, Cork, Ireland; 5 Department of Computational Diagnostic Radiology and Preventive Medicine, The University of Tokyo Hospital, Tokyo, Japan; 6 AAPNA-Institute of Public Health, Jinnah Sindh Medical University, Karachi, Pakistan

**Keywords:** Bangladesh, Cross-sectional, EQ-5D-5 L, Hypertension, Quality-of-life

## Abstract

**Purpose:**

Hypertension is a major health concern in Bangladesh. Assessing the health-related quality of life (HRQoL) among hypertensive patients in Bangladesh can highlight the broad impacts of the condition on morbidity and mortality. Such insights are essential for developing targeted healthcare and prevention strategies to reduce complications, including heart disease, stroke, and kidney failure.

**Methods:**

In this cross-sectional study, 5,086 hypertensive patients aged between 18 and 80 were recruited from 75 pharmacies in Bangladesh. We assessed the participants’ health using the EQ-5D-5 L descriptive system and the EQ-VAS. Utility scores were calculated using the Indian EQ-5D-5 L value set. Regression models were employed to identify factors associated with overall HRQoL and individual health dimensions.

**Results:**

Study participants were mean aged 52 years old, with average systolic and diastolic blood pressures of 140.79 mmHg and 85.98 mmHg, respectively. The average EQ-index and EQ-VAS score were 0.83 and 67.47, respectively. 39% reported difficulties with self-care, 43.5% had mobility problems, 80.6% had pain, and 61.2% had anxiety. HRQoL scores decreased significantly with age, according to the multilevel model. Higher education levels, however, were associated with better HRQoL scores. Male respondents reported fewer problems with mobility, self-care, activity, pain, and anxiety. A decrease in HRQoL scores was observed among older individuals, those without formal education, those in lower quintiles, those unemployed, and those with poor blood pressure control, obesity, or fasting glucose.

**Conclusion:**

Study findings indicate disparities in HRQoL based on age, gender, education, and socioeconomic status, highlighting the need for targeted policy interventions.

**Supplementary Information:**

The online version contains supplementary material available at 10.1007/s11136-025-03912-3.

## Introduction

Hypertension, a major contributor to non-communicable diseases (NCDs), is driving a public health crisis in Bangladesh, exacerbating conditions such as cardiovascular diseases, chronic kidney disease, and more [1]. These conditions collectively contribute to a substantial portion of morbidity and mortality in the Bangladeshi population. Health-related quality of life (HRQoL) is a generic indicator of health and well-being, reflecting an individual’s subjective assessment of their sense of well-being and ability to manage daily life [2]. Previous studies have shown that hypertension negatively impacts the physical and mental well-being of patients through short and long-term complications, physical symptoms, lifestyle changes, and emotional distress, all of which impair HRQoL [3, 4]. Study conducted in Sweden [5], China [6], and Hong Kong [7] have found that individuals with uncontrolled hypertension have lower HRQoL compared to those without hypertension or with controlled hypertension. Thus, understanding the HRQoL of hypertensive patients in Bangladesh is essential for monitoring health and shaping effective interventions that can mitigate the growing burden of NCDs in the country.

Numerous instruments are available to measure HRQoL. EuroQol’s 5 dimensions (EQ-5D) is one of the most implementable and widely used instruments for population health studies. The EQ-5D instruments has two versions: the three-level EQ-5D-3 L and the five-level EQ-5D-5 L. The EQ-5D-5 L is considered the most convenient and valid for measuring HRQoL [8], particularly as the EQ-5D-3 L is prone to a potential ceiling effect [9]. Additionally, the Visual Analogue Scale (EQ-VAS) is an integral component of the EQ-5D-5 L, capturing the respondent’s health status. Despite significant improvements in the health system and a growing emphasis on healthy lifestyles in LMICs including Bangladesh [10], there is still a lack of evidence on HRQoL and its relationship with hypertension. A recent study in Bangladesh showed that gender, occupation, and systemic diseases were correlated with HRQoL [11]. While other studies on HRQoL in Bangladesh have mainly focused on diabetic individuals and visually impaired cataract patients [12, 13]. Another study was conducted in 23 tertiary hospitals evaluated the HRQoL of hypertensive patients using the EQ-5D-3 L instrument [14]. Additionally, studies in neighboring countries including India, Pakistan, Sri Lanka, Thailand, and Indonesia employed both EQ-5D-3 L and EQ-5D-5 L instruments, highlighting the regional importance of understanding HRQoL across various population groups and contexts [15, 16]. Studies conducted in Sri Lanka and Thailand extended this exploration to community-dwelling older individuals [17], while research from India, Indonesia, Sri Lanka, and Thailand assessed HRQoL in the general population [18–26]. While global studies consistently showed that hypertension severely diminishes HRQoL, similar comprehensive assessments in Bangladesh, in particular those using updated and locally relevant data, are lacking.

To gather evidence on HRQoL, we conducted electronic database searches (PubMed, EMBASE, Web of Science, CINHAL) focusing on adult populations in South Asia. This search resulted in 35 studies: 6 from Bangladesh, 5 from India, 3 from Pakistan, 6 from Sri Lanka, 5 from Indonesia, and 10 from Thailand. (Appendix Table S1). The six studies conducted in Bangladesh predominantly concentrated on small sample sizes, hospital-based design, specifically targeting populations with diabetes [13, 27], hypertension [14], pregnant women [28], cataract [29], and socioeconomically disadvantaged groups within certain geographical areas [30]. By shedding light on the HRQoL of hypertensive populations in Bangladesh, this study aims to determine the prevalence of health problems among hypertensive populations in both urban and rural areas of Bangladesh using the EQ-5D-5 L, EQ-VAS, and EQ-index scores. Additionally, the study aimed to explore the relationship between sociodemographic factors and the various dimensions of the EQ-5D-5 L, EQ-VAS, and EQ index scores among hypertensive patients.

## Methods

### Settings

The study was conducted in two districts in Bangladesh: Rangpur and Chuadanga (Appendix Figure S1). According to the Population Census 2022 [31], Bangladesh had 165 million, 31% in rural areas, and 69% in urban areas. Chuadanga district had a population of 1,234,066, with 24% living in urban areas and 76% living in rural areas. In Rangpur, the population was 3,169,615, with 33% living in the urban area and 67% living in the rural. Two selected sampled areas: Pirganj Upazila in Rangpur district had a literacy rate of 50.3%, while Alamdanga Upazila in Chuadanga district had a literacy rate of 44.5%. In Bangladesh, the average household size is around 4, while it is 3.78 in Chuadanga District and 3.80 in Rangpur District [32]. 

### Sampling

Rangpur and Chuadanga districts were purposively selected to represent diverse socio-demographic contexts in Bangladesh, with Rangpur representing northern Bangladesh and Chuadanga representing southwestern Bangladesh. These districts were chosen intentionally rather than randomly to capture variations in socio-demographic and geographic contexts. From each district, one subdistrict was randomly selected—Pirganj in Rangpur and Alamdanga in Chuadanga—to ensure unbiased inclusion of subdistricts within the selected districts. This two-stage process was used to ensure that the districts reflected broader socio-demographic contexts while still allowing for random selection within them.

Initially, 40 pharmacies in rural areas (20 in Alamdanga and 20 in Pirganj) were randomly selected to recruit 3,600 hypertensive adults as part of an ongoing cluster-randomized controlled trial (cRCT) [33]. Then, 1,486 hypertensive adults were sampled separately from 35 pharmacies across both subdistricts to include representation of both urban and rural populations for estimating HRQoL. The urban and rural areas were within each subdistrict; the study was not limited to one urban and one rural subdistrict. This approach ensured representation of both urban and rural populations, addressing the study’s objectives.

### Data collection

Data were collected following the study eligibility criteria: 18 year old or older, have hypertension (defined as systolic blood pressure of 140 mmHg or higher and/or diastolic blood pressures of 90 mmHg or higher, or currently take antihypertensive medication), speak local language, lived in the study area, have a mobile phone, and can give consent. The exclusion criteria were the following: pregnancy or lactation, advanced medical conditions (e.g., cancer, heart failure, chronic obstructive pulmonary disease, end-stage renal disease, advanced neurological disease, etc.), and cognitive or psychiatric problems. Data collection was conducted through face-to-face interviews with a structured questionnaire. The detailed description of data collection is described elsewhere [33]. 

### Outcome variables

The EQ-5D-5 L, developed by the EuroQol group, is a widely used preference based measure (PBM) for HRQoL [34, 35]. It consists of two standardized scales: EQ-5D and EQ-VAS [35, 36]. The EQ-5D-5 L descriptive system, which comprises five dimensions (mobility, self-care, usual activities, pain/discomfort, and anxiety/depression) with five severity levels each, was used to assess the respondent’s HRQoL. The respondents reported their health state on the EQ-5D-5 L descriptive system and the EQ-VAS, based on their overall health on the survey day. On the EQ-VAS, an individual’s self-rated health is scaled from zero to 100, with zero representing “the worst health you can imagine” and 100 representing “the best health you can imagine”. The Bangladeshi version of the EQ-5D-5 L questionnaire was used in this study. We obtained the EQ-5D-5 L health state and EQ-VAS directly from respondents’ self-report questionnaires. The EQ-index score, also known as quality of life score or utility score, was derived from the Indian EQ-5D-5 L value set, as a Bangladeshi value set was not available. For each individual, we calculated their corresponding EQ index score based on their self-reported health states. Utility scores of one and zero indicate perfect health and worst health status respectively.

### Demographic, socioeconomic and health status

HRQoL can be associated with an array of factors at the individual level, the family level, the community level, and the society level [37–46]. Considering previous literature [37–46] and the availability of data, this study included a range of variables, including age, sex, education marital status, body mass index (BMI), blood pressure (both systolic and diastolic), household size, household consumption expenditure, and place of residence. Following the previous literature [47, 48], blood pressure (BP) levels were classified as poor (systolic BP ≥ 140 mmHg or diastolic BP ≥ 90 mmHg), intermediate (systolic BP 120–139 mmHg or diastolic BP 80–89 mmHg or treated to BP < 120/ < 80 mmHg), and ideal (systolic BP < 120 mmHg or diastolic BP < 80 mmHg and without any antihypertensive medication). Weight in kilograms divided by height in meters squared was used to calculate body mass index (BMI). Following previous studies [47, 48], we also divided BMI into three groups - Poor: BMI is 30 or greater; Intermediate: BMI is between 25 and 29.9; and Ideal: BMI is less than 25. Fasting blood glucose (FBG) was categorized as Poor: FBG is 126 mg/dL or greater; Intermediate: FBG is between 100 and 125 mg/dL; and Ideal: FBG is less than 100 mg/dL.^12^ The household expenditure quintile (from the lowest 20% to the highest 20%) was determined following Xu and colleagues [49]. 

### Statistical analysis

Descriptive statistics were used to assess the level of reported health problems, EQ-VAS, and EQ-index scores. This included calculating percentages, means, and standard deviations. The normality of the variables was verified using the Kolmogorov-Smirnov test, and since the variables were normally distributed, a one-way analysis of variance (ANOVA) was conducted to compare the mean HRQoL of respondents for each demographic, socioeconomic and health status variables. The distribution of health states often shows unique characteristics, such as skewed and multimodal patterns, with many individuals reporting perfect health (score of 1) and a gap when transitioning from perfect health to other feasible health states.

Analyzing HRQoL data, such as the EQ-5D index and EQ-VAS, poses challenges due to their bounded nature and clustering at boundary values. Tobit regression and logit-transformed linear regression are two methods commonly used to address these issues. Tobit regression is particularly suitable for censored data with many observations at the upper or lower bounds, such as perfect health, though it assumes normality of the latent variable, which can result in biased estimates near the scale boundaries [50–52]. Logit-transformed linear regression accommodates bounded data without requiring normality, effectively linearizing predictor-outcome relationships, but it complicates coefficient interpretation and requires careful handling of boundary values [52]. Tobit regression is generally better suited for data with significant censoring at the upper bound, while logit-transformed regression is appropriate for bounded data with minimal censoring [50–52]. In our study, we applied both Tobit regression and logit-transformed regression to analyze the EQ-5D index and EQ-VAS scores, respectively. Tobit regression was used for the EQ-5D index due to its censoring at 1 (perfect health), while a generalized linear regression model was used for EQ-VAS to assess its association with risk factors. For comparison, we also conducted logit-transformed regression on the EQ-5D index and EQ-VAS scores and evaluated the differences in results between the models.

Additionally, multilevel logistic regressions were used to determine the associated factors for each health dimension. Each dimension of the EQ-5D-5 L was simplified for the multilevel logistic regression models by using a binary coding system. Following previous studies [53, 54], a score of 0 represented no problems, while any score indicating slight to extreme problems or inability to complete the task was coded as 1. This approach facilitated a straightforward comparison of the presence or absence of issues across the different dimensions. Data management and analysis were performed in Stata MP version 17.

### Ethical approval

The ethical approval of the study was obtained from Hitotsubashi University (Approval Number: 2023D016) and informed consent was collected from the study participants and community pharmacists. We informed the participants that their participation was strictly voluntary and that they could withdraw from the survey at any time without having to give any reason or fearing any repercussions. If a participant is uncomfortable answering any question, they may refrain from doing so. All personal information provided by the participants was kept confidential, ensuring that their identities remained anonymous throughout the study.

## Results

### Study characteristics

The study population is summarized in Tables 1 and 2. The study surveyed 5,086 respondents, with a majority being females (56.8%). Participants’ ages ranged from under 30 (5%) to over 70 (10.2%), and 41% had no formal education while 14% had higher education. Employment was higher among males (87%) compared to females (2%), and 71% of respondents lived in rural areas.

**Table 1:** Anthropometric characteristics of the Study Population, Bangladesh 2023

Variables	Mean (CI)			*p*-value (t-statistic)
	Female (*n* = 2887)	Male (*n* = 2199)	Both sexes (*n* = 5086)	
Age, years	50.95 (50.50-51.39)	53.56 (53.03–54.10)	52.08 (51.73–52.42)	< 0.001
Height (cm)	147.42 (147.12-147.72)	160.19 (159.81-160.57)	152.94 (152.65-153.23)	< 0.001
Weight (kg)	54.74 (54.34–55.14)	62.83 (62.37–63.30)	58.24 (57.92–58.56)	< 0.001
SBP (mmHg)	142.01 (141.24-142.78)	139.19 (138.35-140.02)	140.79 (140.22-141.36)	< 0.001
DBP (mmHg)	86.75 (86.31–87.19)	84.96 (84.48–85.44)	85.98 (85.65–86.30)	< 0.001
FBG (mmol/L)	8.10 (7.95–8.26)	8.08 (7.91–8.25)	8.09 (7.98–8.21)	> 0.100

SBP, Systolic blood pressure; DBP, Diastolic blood pressure; FBG, Fasting blood glucose

**Table 2:** Study characteristics of the study participants in Bangladesh

Variables	Frequency (%)			*p*-value (χ^2^-statistic)
	Female (*n* = 2887)	Male (*n* = 2199)	Both sexes (*n* = 5086)	
**Age**,** years**				< 0.001
≤ 30	156 (5.4)	98 (4.5)	254 (5.0)	
31–39	288 (10.0)	254 (11.6)	542 (10.7)	
40–49	780 (27.0)	404 (18.4)	1184 (23.3)	
50–59	778 (27.0)	620 (28.2)	1398 (27.5)	
60–69	627 (21.7)	562 (25.6)	1189 (23.4)	
≥ 70	258 (8.9)	261 (11.9)	519 (10.2)	
**Education**				< 0.001
No education	1572 (54.5)	518 (23.6)	2090 (41.1)	
Primary	793 (27.5)	558 (25.4)	1351 (26.6)	
Secondary	359 (12.4)	550 (25.0)	909 (17.9)	
Higher	163 (5.7)	573 (26.1)	736 (14.5)	
**Religion**				> 0.100
Muslim	2763 (95.7)	2092 (95.1)	4855 (95.5)	
Non-Muslim	124 (4.3)	107 (4.9)	231 (4.5)	
**Marital status**				< 0.001
Married	2208 (76.5)	2124 (96.6)	4332 (85.2)	
Others (Single/Divorced/ Widowed/Separated)	679 (23.5)	75 (3.4)	754 (14.8)	
**Household size**				< 0.001
1–2	543 (18.8)	239 (10.9)	782 (15.4)	
3–4	1299 (45.0)	1052 (47.8)	2351 (46.2)	
≥ 5	1045 (36.2)	908 (41.3)	1953 (38.4)	
**Occupation**				< 0.001
Not working	2783 (96.4)	57 (2.6)	2840 (55.8)	
Working	64 (2.2)	1908 (86.8)	1972 (38.8)	
Retired	30 (1.0)	190 (8.6)	220 (4.3)	
Others	10 (0.4)	44 (2.0)	54 (1.1)	
**BMI status**				< 0.001
Poor	378 (13.2)	192 (8.8)	570 (11.3)	
Intermediate	986 (34.3)	734 (33.4)	1720 (33.9)	
Ideal	1509 (52.5)	1269 (57.8)	2778 (54.8)	
**Blood pressure status**				< 0.001
Poor	841 (29.1)	503 (22.9)	1344 (26.4)	
Intermediate	1758 (60.9)	1474 (67.0)	3232 (63.6)	
Ideal	288 (10.0)	222 (10.1)	510 (10.0)	
**FBG**				< 0.010
Poor	1340 (46.4)	1116 (50.8)	2456 (48.3)	
Intermediate	993 (34.4)	719 (32.7)	1712 (33.7)	
Ideal	554 (19.2)	364 (16.6)	918 (18.1)	
**Place of residence**				< 0.001
Urban	678 (23.5)	808 (36.7)	1486 (29.2)	
Rural	2209 (76.5)	1391 (63.3)	3600 (70.8)	
**Expenditure quintile**				< 0.001
Quint1 (poorest)	756 (26.2)	306 (13.9)	1062 (20.9)	
Quint2	736 (25.5)	409 (18.6)	1145 (22.5)	
Quint3	517 (17.9)	384 (17.5)	901 (17.7)	
Quint4	504 (17.5)	470 (21.4)	974 (19.2)	
Quint5 (richest)	374 (13.0)	630 (28.7)	1004 (19.7)	

**Blood pressure**: Poor: When SBP ≥ 140 or DBP ≥ 90 mm Hg; Intermediate: When SBP 120–139 or DBP 80–89 or treated BP < 140/<90 mm Hg; Ideal: When BP < 120 and < 80 mm Hg

**BMI**: Poor: When BMI is 30 or greater; Intermediate: When BMI is between 25 and 29.9; Ideal: When BMI is less than 25

**FBG**: Poor: When FBG is 126 mg/dL or greater; Intermediate: When FBG is between 100 and 125 mg/dL; Ideal: When FBG is less than 100 mg/dL

### EQ-5D-5 L outcomes by age and sex

**Fig. 1:**
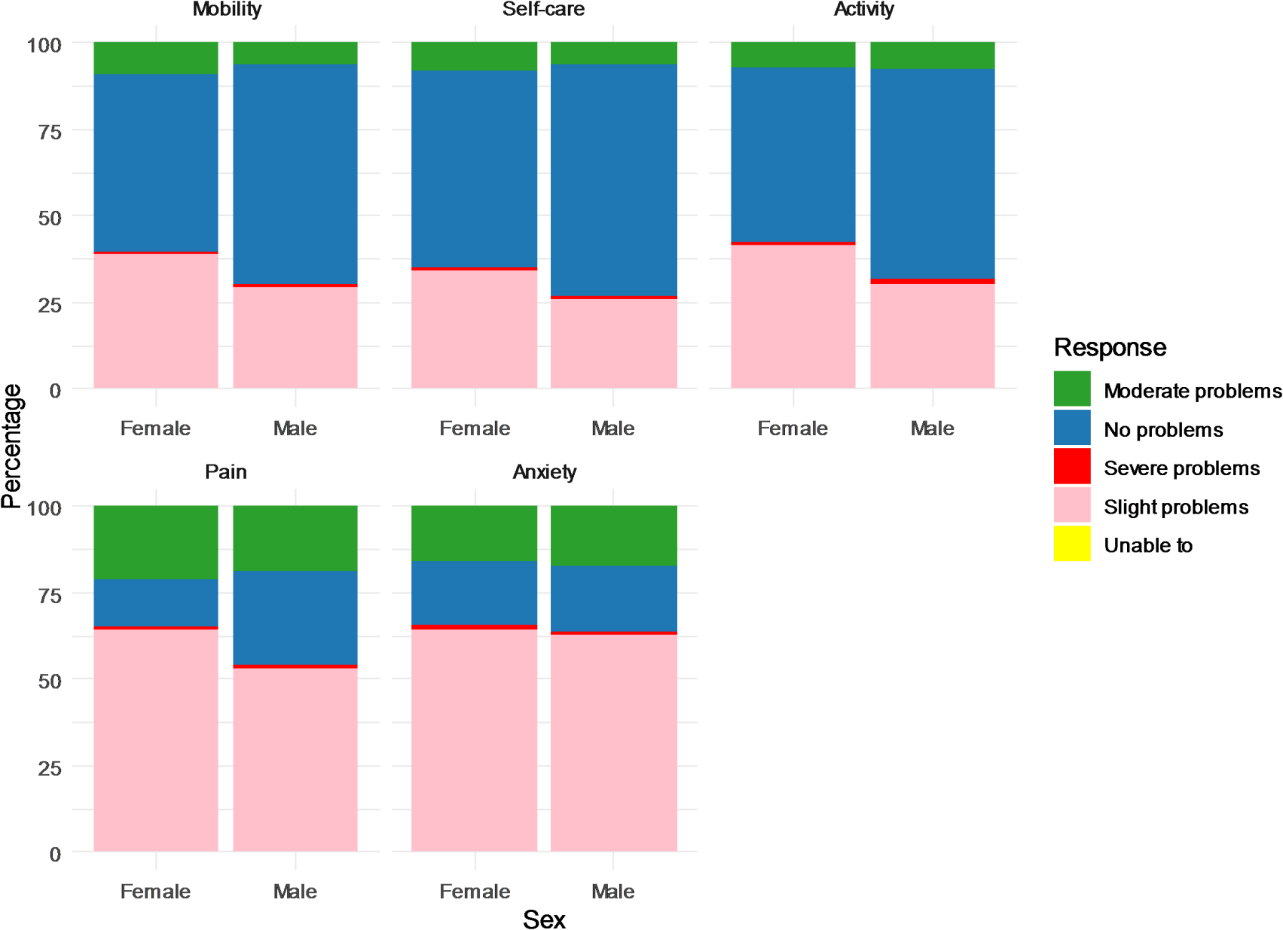
Percentage of reporting any problems in 5 health domains, by sex

Figure 1 illustrates the percentage distribution of five health dimensions among hypertensive population. Self-care issues were the least reported (39.0%), while pain was the most common (80.6%). The detailed information is in the appendix (Appendix Table S2). Mobility (43.5%), usual activities (45%), and anxiety (61.2%) were also relatively high. Females reported higher mobility issues (48.9%) than males (36.3%), but severe mobility problems were more prevalent among males. Significant gender differences were found in mobility, self-care, activity, and pain/discomfort, but not in anxiety/depression (Appendix Table S2). The distribution of reported problems across the EQ-5D-5 L dimensions varies by age and sex, as shown in Table 3 significant differences were found in mobility, self-care, usual activities, pain/discomfort, and anxiety/depression across different age groups for both sexes. Respondents aged 30 years and younger had fewest mobility issues, whereas those above 70 years experienced the highest mobility problems. Similarly, anxiety and depression were most prevalent among respondents aged 60–69. Health issues generally increased with age, except for a decrease in anxiety/depression in those over 70 (Appendix Figure S2). The detailed percentage breakdown of these problems for males and females by age group can be found in the supplemental appendix Tables S3 and S4, respectively.

**Table 3:** Problems in EQ-5D-5 L dimensions by age groups for both sexes, Bangladesh

EQ-5D-5 L dimensions	Age groups (years) (%)						*p*-value (χ^2^-statistic)
	≤ 30 *n* = 254	31–39 *n* = 542	40–49 *n* = 1184	50–59 *n* = 1398	60–69 *n* = 1189	≥ 70 *n* = 519	
**Mobility**							< 0.001
No problems	86.2	78.8	65.1	54.3	45.6	30.3	
Slight problems	12.6	19.0	30.7	38.7	40.5	48.0	
Moderate problems	0.8	2.0	4.1	6.4	12.4	20.0	
Severe problems	0.4	0.2	0.2	0.6	1.4	1.7	
Unable to	0.0	0.0	0.0	0.0	0.2	0.0	
**Self-care**							< 0.001
No problems	87.8	81.4	70.4	58.6	51.2	34.3	
Slight problems	10.2	16.6	25.8	33.5	36.0	45.7	
Moderate problems	1.2	1.9	3.7	7.0	10.8	17.2	
Severe problems	0.8	0.2	0.0	0.9	1.9	2.9	
Unable to	0.0	0.0	0.0	0.1	0.2	0.0	
**Activity**							< 0.001
No problems	84.7	78.4	61.6	52.2	45.2	31.4	
Slight problems	13.8	19.0	34.2	38.6	43.2	50.3	
Moderate problems	1.2	2.2	4.0	8.3	9.0	16.0	
Severe problems	0.0	0.4	0.3	0.9	2.4	2.3	
Unable to	0.4	0.0	0.0	0.1	0.2	0.0	
**Pain**							< 0.001
No problems	51.6	35.2	22.0	16.2	10.6	9.6	
Slight problems	42.5	54.2	60.0	60.7	62.7	59.7	
Moderate problems	5.5	10.0	17.5	21.9	24.6	29.1	
Severe problems	0.4	0.6	0.3	1.1	2.1	1.5	
Unable to	0.0	0.0	0.3	0.1	0.0	0.0	
**Anxiety**							< 0.001
No problems	33.5	24.0	18.7	16.8	15.4	19.3	
Slight problems	59.5	64.2	65.2	63.3	63.8	61.9	
Moderate problems	6.7	11.1	15.0	19.0	19.1	17.2	
Severe problems	0.4	0.7	1.1	0.9	1.5	1.4	
Unable to	0.0	0.0	0.0	0.1	0.2	0.4	

Table 4 shows that the mean of HRQoL, measured by the EQ-5D index score and EQ-VAS index score, varies across socioeconomic characteristics. The overall mean of EQ-5D index is 0.83, while the mean EQ-VAS score is 67.47. Males, younger individuals, those with higher education, married and employed respondents, and urban residents reported on average higher HRQoL. Health indicators like ideal blood pressure and fasting glucose, as well as household expenditure, are also correlated with HRQoL. Table 5 presents an overview of the determinants of HRQoL (EQ-5D index and EQ-VAS scores) from multiple regression models. Age, gender, education, marital status, occupation, place of residence, and expenditure quintile are significantly associated with HRQoL. Specifically, individuals over the age of 30 exhibited lower HRQoL scores compared to those under 30, while respondents with secondary or higher education levels tend to have higher HRQoL scores. Moreover, single or divorced individuals, as well as retired individuals, tend to have lower HRQoL scores. Urban residents generally report higher HRQoL scores compared to their rural counterparts, whereas the richest respondents have lower HRQoL scores compared to the poorest respondents.

**Table 4:** Mean of HRQoL by socio-demographic characteristics in Bangladesh

Variables	EQ-5D index			EQ VAS score	
	Mean (SD)	*p*-value ^a^		Mean (SD)	*p*-value ^a^
**Age**,** years**		< 0.001			< 0.001
≤ 30	0.93 (0.12)			79.88 (13.86)	
31–39	0.90 (0.11)			74.74 (14.38)	
40–49	0.86 (0.12)			69.45 (14.53)	
50–59	0.83 (0.16)			66.89 (14.91)	
60–69	0.79 (0.20)			63.07 (14.50)	
≥ 70	0.76 (0.19)			60.99 (14.42)	
**Gender**		< 0.001			< 0.001
Female	0.82 (0.16)			65.64 (14.42)	
Male	0.85 (0.17)			69.88 (16.15)	
**Education**		< 0.001			< 0.001
No education	0.80 (0.17)			63.62 (13.70)	
Primary	0.84 (0.16)			67.78 (14.90)	
Secondary	0.87 (0.16)			71.45 (15.77)	
Higher	0.88 (0.15)			72.95 (16.92)	
**Religion**		> 0.100			> 0.100
Muslim	0.84 (0.17)			67.47 (15.31)	
Non-Muslim	0.83 (0.15)			67.48 (15.91)	
**Marital status**		< 0.001			< 0.001
Married	0.84 (0.16)			68.49 (15.29)	
Others(Single/Divorced/ Widowed/Separated)	0.79 (0.18)			61.62 (14.25)	
**Household size**		< 0.010			< 0.001
1–2	0.82 (0.16)			64.47 (13.84)	
3–4	0.84 (0.16)			68.10 (15.56)	
≥ 5	0.83 (0.18)			67.92 (15.50)	
**Occupation**		< 0.001			< 0.001
Not working	0.83 (0.16)			65.88 (14.35)	
Working	0.86 (0.16)			70.75 (15.50)	
Retired	0.69 (0.27)			57.52 (18.37)	
Others	0.89 (0.10)			72.04 (15.22)	
**BMI status**		> 0.100			< 0.100
Poor	0.84 (0.18)			67.92 (15.24)	
Intermediate	0.84 (0.17)			68.04 (15.18)	
Ideal	0.83 (0.16)			67.02 (15.46)	
**Blood pressure status**		> 0.100			< 0.001
Poor	0.83 (0.16)			66.33 (14.89)	
Intermediate	0.83 (0.17)			67.65 (15.60)	
Ideal	0.85 (0.16)			69.37 (14.56)	
**FBG**		< 0.100			> 0.100
Poor	0.83 (0.17)			67.41 (15.42)	
Intermediate	0.84 (0.16)			67.53 (15.54)	
Ideal	0.84 (0.17)			67.52 (14.74)	
**Place of residence**		> 0.100			> 0.100
Urban	0.83 (0.20)			67.81 (18.38)	
Rural	0.84 (0.15)			67.33 (13.88)	
**Expenditure quintile**		< 0.010			< 0.001
Quint1 (poorest)	0.82 (0.15)			65.73 (13.18)	
Quint2	0.84 (0.17)			68.00 (13.41)	
Quint3	0.84 (0.16)			68.36 (14.99)	
Quint4	0.85 (0.16)			67.78 (16.08)	
Quint5 (richest)	0.83 (0.20)			67.62 (18.62)	
**Total**	0.83 (0.17)			67.47 (15.33)	

^a^ Note: This is the p-value of the F-statistic. P-value is significant when ≤ 0.05

**Blood pressure**: Poor: When SBP ≥ 140 or DBP ≥ 90 mm Hg; Intermediate: When SBP 120–139 or DBP 80–89 or treated BP < 140/<90 mm Hg; Ideal: When BP < 120 and < 80 mm Hg

**BMI**: Poor: When BMI is 30 or greater; Intermediate: When BMI is between 25 and 29.9; Ideal: When BMI is less than 25

**FBG**: Poor: When FBG is 126 mg/dL or greater; Intermediate: When FBG is between 100 and 125 mg/dL; Ideal: When FBG is less than 100 mg/dL

**Table 5:** Regression analyses on health-related quality of life scores, Bangladesh

Variables	EQ-5D index (Tobit regression)				EQ-VAS score (OLS regression)		
	$\:\varvec{\beta\:}$	95% CI	*p*-values		$\:\varvec{\beta\:}$	95% CI	*p*-values
**Age**,** years**							
≤ 30	0	NA			0	NA	
31–39	-0.03	-0.05– -0.01	**0.01**		-5.67	-7.83 – -3.51	**< 0.01**
40–49	-0.06	-0.08 – -0.04	**< 0.01**		-9.56	-11.56 – -7.56	**< 0.01**
50–59	-0.09	-0.1 – -0.07	**< 0.01**		-11.93	-13.96 – -9.90	**< 0.01**
60–69	-0.11	-0.13– -0.09	**< 0.01**		-15.07	-17.18 – -12.95	**< 0.01**
≥ 70	-0.14	-0.17– -0.12	**< 0.01**		-16.30	-18.68 – -13.93	**< 0.01**
**Gender**							
Female	0	NA			0	NA	
Male	0.02	-0.00–0.05	0.06		3.70	1.42–5.99	**< 0.01**
**Education**							
No education	0	NA			0	NA	
Primary	0.01	0.00–0.02	**0.03**		1.88	0.82–2.93	**< 0.01**
Secondary	0.04	0.02–0.05	**< 0.01**		3.98	2.69–5.28	**< 0.01**
Higher	0.04	0.03–0.06	**< 0.01**		5.39	3.91–6.87	**< 0.01**
**Religion**							
Muslim	0	NA			0	NA	
Non-Muslim	-0.00	-0.02–0.02	0.71		0.37	-1.51–2.25	0.70
**Marital status**							
Married	0	NA			0	NA	
Others (Single/Divorced/ Widowed/Separated)	-0.01	-0.03– -0.00	**0.03**		-2.23	-3.46 – -1.00	**< 0.01**
**Household size**							
1–2	0	NA			0	NA	
3–4	-0.01	-0.03–0.00	0.06		-0.45	-1.71–0.81	0.48
≥ 5	-0.01	-0.02–0.00	0.13		0.57	-0.67–1.80	0.37
**Occupation**							
Not working	0	NA			0	NA	
Working	0.01	-0.01–0.03	0.46		0.36	-1.95–2.68	0.76
Retired	-0.11	-0.14– -0.08	**< 0.01**		-8.34	-11.20 – -5.49	**< 0.01**
Others	0.03	-0.01–0.08	0.16		1.90	-2.32–6.12	0.38
**BMI status**							
Poor	0	NA			0	NA	
Intermediate	0.00	-0.01–0.02	0.75		0.62	-0.73–1.97	0.37
Ideal	0.00	-0.01–0.02	0.47		0.70	-0.60–2.00	0.29
**Blood pressure status**							
Poor	0	NA			0	NA	
Intermediate	-0.00	-0.01–0.01	0.77		0.44	-0.47–1.36	0.34
Ideal	0.01	-0.01–0.02	0.47		1.38	-0.08–2.83	0.06
**FBG**							
Poor	0	NA			0	NA	
Intermediate	0.01	-0.00–0.01	0.28		-0.08	-0.96–0.81	0.87
Ideal	0.01	-0.00–0.02	0.21		-0.59	-1.69–0.51	0.29
**Place of residence**							
Urban	0	NA			0	NA	
Rural	0.01	0.00–0.02	**0.02**		1.16	0.13–2.20	**0.03**
**Expenditure quintile**							
Quint1 (poorest)	0	NA			0	NA	
Quint2	0.00	-0.01–0.02	0.51		0.74	-0.51–1.98	0.25
Quint3	0.01	-0.01–0.02	0.35		0.23	-1.08–1.53	0.73
Quint4	0.00	-0.01–0.02	0.49		-1.35	-2.68 – -0.02	**0.05**
Quint5 (richest)	-0.02	-0.04– -0.01	**0.01**		-3.30	-4.78 – -1.82	**< 0.01**
R^2^ Nagelkerke	-0.101				0.156		

Others refers to single/Divorced/widowed/Separated); BMI, body mass index; FBG, fasting blood glucose

**Blood pressure status**: Poor: When SBP ≥ 140 or DBP ≥ 90 mm Hg; Intermediate: When SBP 120–139 or DBP 80–89 or treated BP < 140/<90 mm Hg; Ideal: When BP < 120 and < 80 mm Hg

**BMI**: Poor: When BMI is 30 or greater; Intermediate: When BMI is between 25 and 29.9; Ideal: When BMI is less than 25

**FBG**: Poor: When FBG is 126 mg/dL or greater; Intermediate: When FBG is between 100 and 125 mg/dL; Ideal: When FBG is less than 100 mg/dL

The logit-transformed regression model results, presented in the supplemental appendix (Table S5), highlight age and education as the most significant predictors of health-related quality of life, with older age groups showing consistently lower EQ-5D index and EQ-VAS scores compared to younger individuals, and higher education levels being associated with better scores. Males reported significantly higher scores than females, and rural residence was linked to slightly better EQ-VAS scores but no significant difference in the EQ-5D index. Retired individuals exhibited notably lower scores across both measures compared to those not working, while marital status impacted only EQ-VAS scores, with single, divorced, widowed, or separated individuals reporting lower scores than married individuals. Larger household sizes were associated with marginally lower EQ-5D index scores, but this relationship was not significant for EQ-VAS scores. Expenditure quintiles showed a mixed pattern, with middle-income groups generally having better EQ-5D scores, whereas the richest quintile had significantly lower EQ-VAS scores compared to the poorest. BMI status, blood pressure, and fasting glucose levels did not significantly influence either health-related quality of life measure.

Table 6 presents the results of multilevel logistic regression models for each dimension of the EQ-5D-5 L. The results of the multilevel logistic regression models demonstrated a consistent pattern in the association between age, gender, education level, and residential area with the likelihood of experiencing problems across various dimensions of HRQoL. Specifically, individuals aged 31–39 years and above, male respondents, those with lower education levels, single or divorced individuals, and rural residents were more likely to report problems in mobility, self-care, activity, pain, and anxiety compared to their respective counterparts. Additionally, rural residents were more likely than urban residents to report problems across multiple dimensions of HRQoL.

**Table 6:** Multilevel logistic regression analyses on reported health problems, Bangladesh, 2023

Variables	Odds ratio (95% CI)				
	Mobility	Self-care	Activity	Pain	Anxiety
Age					
≤ 30	1.00	1.00	1.00	1.00	1.00
31–39	1.66 (1.07–2.58)	1.88 (1.18–2.98)	1.81 (1.17–2.78)	2.87 (2.01–4.11)	1.66 (1.16–2.38)
40–49	2.61 (1.74–3.91)	2.80 (1.82–4.30)	3.35 (2.25–4.98)	4.76 (3.38–6.69)	2.22 (1.58–3.12)
50–59	4.21 (2.80–6.32)	4.66 (3.03–7.16)	4.74 (3.18–7.08)	7.48 (5.24–10.69)	2.35 (1.66–3.33)
60–69	5.29 (3.49–8.03)	5.78 (3.72–8.98)	5.75 (3.81–8.68)	12.00 (8.12–17.73)	2.31 (1.59–3.34)
≥ 70	9.52 (6.04-15.00)	10.70 (6.67–17.18)	9.60 (6.13–15.03)	13.75 (8.59–22.02)	1.67 (1.09–2.53)
**Gender**					
Female	1.00	1.00	1.00	1.00	1.00
Male	1.19 (0.81–1.76)	0.94 (0.64–1.39)	0.87 (0.59–1.27)	0.56 (0.36–0.88)	0.72 (0.46–1.14)
**Education**					
No education	1.73 (1.35–2.23)	1.97 (1.52–2.54)	2.45 (1.91–3.14)	2.10 (1.59–2.79)	1.56 (1.18–2.07)
Primary	1.57 (1.23–1.99)	1.59 (1.24–2.04)	1.81 (1.43–2.30)	2.04 (1.57–2.64)	1.51 (1.16–1.96)
Secondary	1.15 (0.90–1.48)	1.00 (0.78–1.30)	1.24 (0.97–1.58)	1.54 (1.20–1.98)	1.09 (0.84–1.41)
Higher	1.00	1.00	1.00	1.00	1.00
**Religion**					
Muslim	1.00	1.00	1.00	1.00	1.00
Non-Muslim	1.01 (0.74–1.37)	1.01 (0.74–1.38)	1.10 (0.81–1.50)	1.37 (0.90–2.08)	1.11 (0.77–1.60)
**Marital status**					
Married	1.00	1.00	1.00	1.00	1.00
Others (Single/Divorced/ Widowed/Separated)	1.13 (0.93–1.38)	1.37 (1.13–1.66)	1.32 (1.09–1.60)	1.00 (0.75–1.33)	1.71 (1.32–2.23)
**Household size**					
1–2	1.00	1.00	1.00	1.00	1.00
3–4	1.10 (0.91–1.34)	1.30 (1.07–1.58)	1.09 (0.90–1.32)	1.04 (0.79–1.37)	0.92 (0.71–1.19)
≥ 5	1.00 (0.83–1.22)	1.22 (1.01–1.48)	1.07 (0.89–1.30)	1.02 (0.78–1.34)	0.67 (0.52–0.85)
**Occupation**					
Not working	1.00	1.00	1.00	1.00	1.00
Working	0.47 (0.32–0.69)	0.67 (0.45–0.99)	0.74 (0.50–1.09)	0.63 (0.40-1.00)	1.49 (0.94–2.37)
Retired	2.14 (1.33–3.43)	3.01 (1.86–4.89)	2.94 (1.81–4.79)	1.41 (0.73–2.71)	2.97 (1.58–5.57)
Others	0.44 (0.21–0.89)	0.60 (0.28–1.25)	0.66 (0.33–1.34)	0.50 (0.24–1.04)	2.47 (0.98–6.24)
**Place of residence**					
Urban	1.00	1.00	1.00	1.00	1.00
Rural	3.10 (2.29–4.20)	1.27 (0.92–1.74)	1.19 (0.86–1.65)	1.00 (0.65–1.53)	1.06 (0.71–1.58)
**Expenditure quintile**					
Quint1 (poorest)	0.93 (0.72–1.19)	1.22 (0.95–1.57)	0.92 (0.72–1.18)	0.83 (0.60–1.13)	0.75 (0.56–1.01)
Quint2	0.82 (0.64–1.04)	0.91 (0.72–1.17)	0.82 (0.65–1.04)	0.75 (0.57-1.00)	0.79 (0.59–1.05)
Quint3	0.98 (0.77–1.25)	0.98 (0.76–1.25)	0.81 (0.64–1.03)	0.70 (0.52–0.93)	0.74 (0.56–0.98)
Quint4	1.07 (0.85–1.34)	1.00 (0.80–1.26)	0.95 (0.77–1.19)	0.76 (0.59–0.98)	1.02 (0.78–1.33)
Quint5 (richest)	1.00	1.00	1.00	1.00	1.00

Adjusted variables: BMI status, current blood pressure status, and fasting glucose level

## Discussion

The primary aim of this study was to assess HRQoL and the prevalence of health problems among hypertensive populations in both urban and rural areas of Bangladesh, utilizing the EQ-5D-5 L, EQ-VAS, and EQ-index scores. Our findings indicated that men reported severer mobility issues than women (48.9% versus 36.3%), and HRQoL scores, as measured by the EQ-index and EQ-VAS, significantly decreased with age. Notably, higher educational levels were associated with improved HRQoL scores. In examining gender differences, male respondents exhibited lower odds of experiencing problems related to mobility, self-care, activity, pain, and anxiety compared to female respondents. HRQoL scores were notably lower among older individuals, those without formal education, individuals in the lower socioeconomic quintiles, the unemployed, and those with poor fasting glucose levels.

Our study indicated that among the five EQ-5D dimensions, the most frequently reported problem was anxiety/depression which was consistent with other studies in Bangladesh [13, 29]. Nonetheless, other studies have identified the pain/discomfort dimension as the most common health problem [15–21, 24–26, 28, 55]. Conversely, another study reported that usual activities were the highest reported problem [56]. In our study, the least reported problem was the self-care dimension, consistent with other studies [13, 16–21, 24–26, 28, 29]. It is challenging to compare HRQoL results across studies due to varying perceptions of life conditions, cultural differences, health system performance, and other socio-demographic circumstances [57, 58]. Additionally, we found that younger participants had a higher prevalence of anxiety/depression (66.5%), which was also found by a recent study [13]. Our study also demonstrated that the percentage of individuals reporting problems in the five dimensions increased with age, which was corroborated by other research from Sri Lanka [21]. Further, our study showed that male respondents consistently reported better HRQoL than female respondents, which were found in a number of existing studies [13, 16, 17, 24, 30, 59]. 

The variations in HRQoL across demographic groups may be attributed to several factors such as gender-based disparities in access to healthcare, social support, and socioeconomic status, along with higher rates of chronic illness and psychological stress commonly reported among female respondents. This variation also may be because of the routine family duties, lower health knowledge, and limited access to healthcare resources being partially responsible for the lower HRQoL among our female respondents. Moreover, married respondents showed better HRQoL than those currently not married, this result was also supported by previous research [17]. This may occur because married respondents might have better social support, emotional security, and shared resources, which improve their well-being. However, several studies have suggested that never-married respondents have better HRQoL than married ones [24, 30]. This may be because never-married respondents may benefit from greater autonomy, fewer caregiving duties, and less relationship-related stress, contributing to better HRQoL. Differences in age, financial independence, and social norms across populations can also play a significant role in shaping these outcomes. Our study also found that as educational qualifications increased, HRQoL scores improved, this finding was supported by several other studies [16, 17, 23, 24, 30]. This might happen as a result of individuals with higher levels of education having better HRQoL because they have better access to healthcare, are more health-literate, and have an improved socioeconomic status- all of which increase general well-being and health outcomes. Conversely, another study among Bangladeshi pregnant women indicated that HRQoL decreased as their educational level increased [28]. This may occur due to higher expectations and greater awareness of potential health risks.

Urban residents reported higher HRQoL than rural residents, which is consistent with several other studies [23, 24, 59]. Conversely, another study found the opposite, reporting better HRQoL among rural residents [13]. The difference may occur due to varying factors influencing the quality of life in different settings. Urban residents often have better access to healthcare facilities, education, and employment opportunities, which can contribute to a higher HRQoL. However, the stressors associated with urban life, such as pollution, overcrowding, and a fast-paced lifestyle, might diminish the perceived quality of life for some individuals. On the other hand, rural residents may experience a better HRQoL due to a more relaxed environment, stronger community ties, and lower living costs. Nonetheless, limited access to healthcare, education, and economic opportunities can negatively impact HRQoL in rural areas. Our study also showed that currently working respondents showed better HRQoL than those not working, this is also supported by several studies [16, 17, 24, 59]. This may occur as currently employed respondents might show better HRQoL due to increased economic stability, a sense of purpose, and social relations that come with employment, all of which exhibit positive mental and physical well-being.

Our study found an average EQ-index of 0.83 among the hypertensive population in Bangladesh, with an EQ-VAS of 67.47. Comparatively, Bhutan’s general population had a higher EQ-index of 0.95, while Indonesia’s diabetic patients had an index of 0.86 [60]. India’s general population averaged 0.8431 [18], older adults in Thai community dwellings had 0.815 [17], and Sri Lankan patients with chronic renal disease had the lowest at 0.53 [16]. These variations stem from differences in health status, financial conditions, healthcare access, cultural practices, lifestyle, and age distribution. Our findings indicate that socioeconomic factors play a crucial role in HRQoL in Bangladesh. Higher education levels, employment, and urban residency are linked to better HRQoL, emphasizing the need for socioeconomic development. To enhance overall well-being, a comprehensive healthcare strategy addressing both socioeconomic factors and health indicators like blood pressure and glucose levels is essential.

Our study has several strengths. This study is the first to use the EQ-5D-5 L instrument to assess HRQoL among the hypertensive population in Bangladesh, utilizing a large representative sample. Employing validated tools such as the EQ-5D-5 L, EQ-VAS, and EQ-index scores, the study ensures robust and reliable data collection. By including both urban and rural populations, the study offers a comprehensive view of HRQoL across different geographical areas, making the findings more generalizable. By focusing on a developing country context, the research adds valuable knowledge to the limited literature on HRQoL in low- and middle-income countries and offers crucial policy implications for improving healthcare strategies in Bangladesh.

To interpret the results cautiously, despite several strengths, a few limitations need to be acknowledged. The cross-sectional design limits the ability to establish causality or track changes in HRQoL over time. The results of our study may not be generalizable to the general population of Bangladesh since we sampled hypertensive people aged 15 years and older. Additionally, the study did not account for potential confounding variables, such as other chronic conditions, and did not explore how diverse cultural factors might influence HRQoL expressions [61]. Although we employed a multistage sampling approach, the combination of purposive and random sampling at different stages posed challenges in calculating and applying accurate survey sampling weights. Consequently, the analyses presented in this study are unweighted. While this mixed sampling method does not compromise the internal validity of our findings, it may limit the generalizability of the results to the general population. To address this limitation, we recommend that future studies adopt fully probabilistic sampling methods to enable the calculation and application of survey weights, ensuring more representative results. Finally, the study used a value set that was obtained in India because one for Bangladesh was not available. These limitations highlight the need for further research with longitudinal data and more diverse samples. Besides, a more accurate understanding of HRQoL can be obtained by comparing the hypertensive population to a non-hypertensive or healthy population.

## Conclusion and policy implications

Improving HRQoL among hypertensive populations in Bangladesh requires targeted health interventions, community-based counseling programs, and tailored support for vulnerable demographic groups, particularly older individuals, those with lower education levels, and women. This study highlights significantly lower HRQoL among hypertensive individuals across various disadvantaged groups, underscoring the urgent need for practical and impactful policy actions. To tackle this issue, several measures are suggested. First, nationwide awareness campaigns and community-based screenings should be implemented to promote early detection and timely management of hypertension. Strengthening primary healthcare systems is essential, including ensuring consistent access to essential medications and providing healthcare professionals with updated training. Encouraging healthier lifestyles—such as reducing salt intake, increasing physical activity, and promoting balanced diets—through targeted community programs can yield substantial improvements in outcomes. Engaging community health workers particularly community pharmacies to support hypertension management and incorporating mental health services into care delivery can address both the physical and emotional challenges faced by hypertensive individuals. Finally, establishing robust systems for monitoring and evaluating these initiatives will ensure their effectiveness and allow for ongoing improvements.

By implementing these measures, policymakers can enhance the HRQoL for hypertensive individuals and reduce the broader burden of heart disease in Bangladesh, contributing to a healthier and more equitable society.

## Electronic supplementary material

Below is the link to the electronic supplementary material.

Supplementary Material 1

## References

[1] Noncommunicable Diseases [https://www.who.int/news-room/fact-sheets/detail/noncommunicable-diseases]

[2] Bowling, A. (1997). Measuring health: a review of quality of life measurement scales.

[3] Sang, S., Kang, N., Liao, W., Wu, X., Hu, Z., Liu, X., Wang, C., & Zhang, H. (2021). The influencing factors of health-related quality of life among rural hypertensive individuals: A cross-sectional study. *Health and Quality of life Outcomes*, *19*, 1–10. 34663349 10.1186/s12955-021-01879-6 PMC8524889

[4] Katsi, V., Kallistratos, M. S., Kontoangelos, K., Sakkas, P., Souliotis, K., Tsioufis, C., Nihoyannopoulos, P., Papadimitriou, G. N., & Tousoulis, D. (2017). Arterial hypertension and health-related quality of life. *Frontiers in Psychiatry*, *8*, 270. 29255431 10.3389/fpsyt.2017.00270 PMC5722974

[5] Bardage, C., & Isacson, D. G. (2001). Hypertension and health-related quality of life: An epidemiological study in Sweden. *Journal of Clinical Epidemiology*, *54*(2), 172–181. 11166533 10.1016/s0895-4356(00)00293-6

[6] Xiao, M., Zhang, F., Xiao, N., Bu, X., Tang, X., & Long, Q. (2019). Health-related quality of life of hypertension patients: A population-based cross-sectional study in Chongqing, China. *International Journal of Environmental Research and Public Health*, *16*(13), 2348. 31277210 10.3390/ijerph16132348 PMC6652141

[7] Wong, E. L. Y., Xu, R. H., & Cheung, A. W. L. (2019). Health-related quality of life among patients with hypertension: Population-based survey using EQ-5D-5L in Hong Kong SAR, China. *BMJ open*, *9*(9), e032544. 31562165 10.1136/bmjopen-2019-032544 PMC6773333

[8] [https://euroqol.org/information-and-support/euroqol-instruments/eq-5d-5l/].

[9] Devlin, N. J., & Brooks, R. (2017). EQ-5D and the EuroQol group: Past, present and future. *Applied Health Economics and Health Policy*, *15*, 127–137. 28194657 10.1007/s40258-017-0310-5 PMC5343080

[10] Islam, S. M. S., Uddin, R., Das, S., Ahmed, S. I., Zaman, S. B., Alif, S. M., Hossen, M. T., Sarker, M., Siopis, G., & Livingstone, K. M. (2023). The burden of diseases and risk factors in Bangladesh, 1990–2019: A systematic analysis for the global burden of Disease Study 2019. *The Lancet Global Health*, *11*(12), e1931–e1942. 37973341 10.1016/S2214-109X(23)00432-1 PMC10664824

[11] Sarker, A. R. (2021). Health-related quality of life among older citizens in Bangladesh. *SSM-Mental Health*, *1*, 100031.

[12] Shetty, A., Afroz, A., Ali, L., Siddiquea, B. N., Sumanta, M., & Billah, B. (2021). Health-related quality of life among people with type 2 diabetes mellitus–A multicentre study in Bangladesh. *Diabetes & Metabolic Syndrome: Clinical Research & Reviews*, *15*(5), 102255. 10.1016/j.dsx.2021.102255 34479101

[13] Barua, L., Faruque, M., Chowdhury, H. A., Banik, P. C., & Ali, L. (2021). Health-related quality of life and its predictors among the type 2 diabetes population of Bangladesh: A nation-wide cross-sectional study. *J Diabetes Investig*, *12*(2), 277–285. 32564501 10.1111/jdi.13331 PMC7858106

[14] Mannan, A., Akter, K. M., Akter, F., Chy, N. U. H. A., Alam, N., Pinky, S. D., Chowdhury, A. F. M. N., Biswas, P., Chowdhury, A. S., & Hossain, M. A. (2022). Association between comorbidity and health-related quality of life in a hypertensive population: A hospital-based study in Bangladesh. *Bmc Public Health*, *22*(1), 181. 35081905 10.1186/s12889-022-12562-w PMC8793199

[15] Muhammed, H., Goyal, M., Lal, V., Singh, S., & Dhir, V. (2018). Neuropsychiatric manifestations are not uncommon in Indian lupus patients and negatively affect quality of life. *Lupus*, *27*(4), 688–693. 29241417 10.1177/0961203317747720

[16] Kularatna, S., Senanayake, S., Gunawardena, N., & Graves, N. (2019). Comparison of the EQ-5D 3L and the SF-6D (SF-36) contemporaneous utility scores in patients with chronic kidney disease in Sri Lanka: A cross-sectional survey. *British Medical Journal Open*, *9*(2), e024854. 10.1136/bmjopen-2018-024854 PMC6398797 30772857

[17] Aung, T. N. N., Moolphate, S., Koyanagi, Y., Angkurawaranon, C., Supakankunti, S., Yuasa, M., & Aung, M. N. (2022). Determinants of Health-Related Quality of Life among Community-Dwelling Thai older adults in Chiang Mai, Northern Thailand. *Risk Manag Healthc Policy*, *15*, 1761–1774. 36164477 10.2147/RMHP.S370353 PMC9508892

[18] Jyani, G., Prinja, S., Garg, B., Kaur, M., Grover, S., Sharma, A., & Goyal, A. (2023). Health-related quality of life among Indian population: The EQ-5D population norms for India. *J Glob Health*, *13*, 04018. 36799239 10.7189/jogh.13.04018 PMC9936451

[19] Purba, F. D., Hunfeld, J. A. M., Iskandarsyah, A., Fitriana, T. S., Sadarjoen, S. S., Ramos-Goni, J. M., Passchier, J., & Busschbach, J. J. V. (2017). The Indonesian EQ-5D-5L Value Set. *Pharmacoeconomics*, *35*(11), 1153–1165. 28695543 10.1007/s40273-017-0538-9 PMC5656740

[20] Kularatna, S., Whitty, J. A., Johnson, N. W., Jayasinghe, R., & Scuffham, P. A. (2015). Valuing EQ-5D health states for Sri Lanka. *Quality of Life Research*, *24*(7), 1785–1793. 25543271 10.1007/s11136-014-0906-2

[21] Kularatna, S., Whitty, J. A., Johnson, N. W., Jayasinghe, R., & Scuffham, P. A. (2014). EQ-5D-3L derived population norms for health related quality of life in Sri Lanka. *PLoS One*, *9*(11), e108434. 25365171 10.1371/journal.pone.0108434 PMC4217723

[22] Janssen, M. F., Szende, A., Cabases, J., Ramos-Goni, J. M., Vilagut, G., & Konig, H. H. (2019). Population norms for the EQ-5D-3L: A cross-country analysis of population surveys for 20 countries. *The European Journal of Health Economics*, *20*(2), 205–216. 29445941 10.1007/s10198-018-0955-5 PMC6438939

[23] Kaikeaw, S., Punpuing, S., Chamchan, C., & Prasartkul, P. (2023). Socioeconomic inequalities in health outcomes among Thai older population in the era of Universal Health Coverage: Trends and decomposition analysis. *Int J Equity Health*, *22*(1), 144. 37533003 10.1186/s12939-023-01952-0 PMC10399069

[24] Kangwanrattanakul, K., & Krageloh, C. U. (2024). EQ-5D-3L and EQ-5D-5L population norms for Thailand. *Bmc Public Health*, *24*(1), 1108. 38649833 10.1186/s12889-024-18391-3 PMC11036570

[25] Kangwanrattanakul, K., & Parmontree, P. (2020). Psychometric properties comparison between EQ-5D-5L and EQ-5D-3L in the general Thai population. *Quality of Life Research*, *29*(12), 3407–3417. 32780315 10.1007/s11136-020-02595-2

[26] Pattanaphesaj, J., Thavorncharoensap, M., Ramos-Goni, J. M., Tongsiri, S., Ingsrisawang, L., & Teerawattananon, Y. (2018). The EQ-5D-5L valuation study in Thailand. *Expert Rev Pharmacoecon Outcomes Res*, *18*(5), 551–558. 29958008 10.1080/14737167.2018.1494574

[27] Namdeo, M. K., Verma, S., Das Gupta, R., Islam, R., Nazneen, S., & Rawal, L. B. (2023). Depression and health-related quality of life of patients with type 2 diabetes attending tertiary level hospitals in Dhaka, Bangladesh. *Glob Health Res Policy*, *8*(1), 43. 37845742 10.1186/s41256-023-00328-9 PMC10577997

[28] Mahumud, R. A., Ali, N., Sheikh, N., Akram, R., Alam, K., Gow, J., Sarker, A. R., & Sultana, M. (2019). Measuring perinatal and postpartum quality of life of women and associated factors in semi-urban Bangladesh. *Quality of Life Research*, *28*(11), 2989–3004. 31312976 10.1007/s11136-019-02247-0

[29] Polack, S., Eusebio, C., Mathenge, W., Wadud, Z., Mamunur, A. K., Fletcher, A., Foster, A., & Kuper, H. (2010). The impact of cataract surgery on health related quality of life in Kenya, the Philippines, and Bangladesh. *Ophthalmic Epidemiology*, *17*(6), 387–399. 21090912 10.3109/09286586.2010.528136

[30] Sultana, M., Sarker, A. R., Mahumud, R. A., Ahmed, S., Ahmed, W., Chakrovorty, S., Rahman, H., Islam, Z., & Khan, J. A. (2016). Inequalities in Health Status from EQ-5D findings: A cross-sectional study in Low-Income communities of Bangladesh. *Int J Health Policy Manag*, *5*(5), 301–308. 27239879 10.15171/ijhpm.2016.06 PMC4851999

[31] Bangladesh Bureau of Statistics (BBS). (2022). *Population & Housing Census 2022, Preleminary report*. Dhaka, Bangladesh. In.

[32] Bangladesh Bureau of Statistics (BBS) (2020). Poverty Maps Of Bangladesh 2016, Key Findings. In.

[33] Rahman, M. M., Nakamura, R., Islam, M. M., Alam, M. A., Azmat, S. K., & Sato, M. (2024). Effectiveness of a community pharmacy-based health promotion program on hypertension in bangladesh and pakistan: study protocol for a cluster-randomized controlled trial. *Healthcare*, *12*(14), 1402. 10.3390/healthcare12141402 PMC11276715 39057545

[34] Group, T. E. (1990). EuroQol-a new facility for the measurement of health-related quality of life. *Health Policy*, *16*(3), 199–208. 10109801 10.1016/0168-8510(90)90421-9

[35] Xu, R. H., Sun, R., Tian, L., Cheung, A. W., & Wong, E. L. (2024). Health-related quality of life in primary care patients: A comparison between EQ-5D-5L utility score and EQ-visual analogue scale. *Health and Quality of Life Outcomes*, *22*(1), 2. 38172916 10.1186/s12955-023-02215-w PMC10765691

[36] Snowdon, D. A., Collyer, T. A., Marsh, L., Srikanth, V., Beare, R., Baber, S., Naude, K., & Andrew, N. E. (2024). Healthcare consumer acceptability of routine use of the EQ-5D-5L in clinical care: A cross-sectional survey. *Quality of Life Research*, 1–15. 10.1007/s11136-024-03598-z PMC11045645 38321194

[37] Chen, C., Liu, G., Shi, Q., Sun, Y., Zhang, H., Wang, M., Jia, H., Zhao, Y., & Yao, Y. (2020). Health-related quality of life and associated factors among oldest-old in China. *The Journal of Nutrition Health and Aging*, *24*(3), 330–338. 10.1007/s12603-020-1327-2 PMC7064459 32115616

[38] Durch, J. S., Bailey, L. A., & Stoto, M. A. (1997). Improving health in the community: a role for performance monitoring. 25121202

[39] Glouberman, S., & Millar, J. (2003). Evolution of the determinants of health, health policy, and health information systems in Canada. *American Journal of Public Health*, *93*(3), 388–392. 12604478 10.2105/ajph.93.3.388 PMC1447749

[40] Yao, Q., Liu, C., Zhang, Y., & Xu, L. (2019). Changes in health-related quality of life of Chinese populations measured by the EQ-5D-3 L: A comparison of the 2008 and 2013 National Health services surveys. *Health and Quality of life Outcomes*, *17*, 1–12. 30866953 10.1186/s12955-019-1109-x PMC6417242

[41] Becker, J., Bose-O’Reilly, S., Shoko, D., Singo, J., & Steckling-Muschack, N. (2020). Comparing the self-reported health-related quality of life (HRQoL) of artisanal and small-scale gold miners and the urban population in Zimbabwe using the EuroQol (EQ-5D-3L + C) questionnaire: A cross-sectional study. *Health and Quality of life Outcomes*, *18*, 1–13. 32727498 10.1186/s12955-020-01475-0 PMC7390189

[42] Deng, X., Dong, P., Zhang, L., Tian, D., Zhang, L., Zhang, W., Li, L., Deng, J., Ning, P., & Hu, G. (2017). Health-related quality of life in residents aged 18 years and older with and without disease: Findings from the first provincial health services survey of Hunan, China. *BMJ open*, *7*(9), e015880. 28871016 10.1136/bmjopen-2017-015880 PMC5588974

[43] Alefishat, E., Jarab, S., & Abu Farha, A. (2020). Factors affecting health-related quality of life among hypertensive patients using the EQ‐5D tool. *International Journal of Clinical Practice*, *74*(9), e13532. 32416003 10.1111/ijcp.13532

[44] Jyani, G., Prinja, S., Garg, B., Kaur, M., Grover, S., Sharma, A., & Goyal, A. (2023). Health-related quality of life among Indian population: The EQ-5D population norms for India. *Journal of Global Health*, 13. 10.7189/jogh.13.04018 10.7189/jogh.13.04018 PMC9936451 36799239

[45] Nguyen, L. H., Tran, B. X., Hoang Le, Q. N., Tran, T. T., & Latkin, C. A. (2017). Quality of life profile of general Vietnamese population using EQ-5D-5L. *Health and Quality of life Outcomes*, *15*, 1–13. 29020996 10.1186/s12955-017-0771-0 PMC5637080

[46] Saleh, F., Ara, F., Mumu, S. J., & Hafez, M. A. (2015). Assessment of health-related quality of life of Bangladeshi patients with type 2 diabetes using the EQ-5D: A cross-sectional study. *BMC Research Notes*, *8*, 1–8. 26420245 10.1186/s13104-015-1453-9 PMC4588249

[47] Pengpid, S., & Peltzer, K. (2023). Trends in behavioral and biological risk factors for non-communicable diseases among adults in Bhutan: Results from cross-sectional surveys in 2007, 2014, and 2019. *Frontiers in Public Health*, *11*, 1192183. 37593725 10.3389/fpubh.2023.1192183 PMC10430069

[48] Huffman, M. D., Capewell, S., Ning, H., Shay, C. M., Ford, E. S., & Lloyd-Jones, D. M. (2012). Cardiovascular health behavior and health factor changes (1988–2008) and projections to 2020: Results from the National Health and Nutrition Examination Surveys. *Circulation*, *125*(21), 2595–2602. 22547667 10.1161/CIRCULATIONAHA.111.070722 PMC3914399

[49] Xu, K., Evans, D. B., Kawabata, K., Zeramdini, R., Klavus, J., & Murray, C. J. (2003). Household catastrophic health expenditure: A multicountry analysis. *Lancet*, *362*(9378), 111–117. 12867110 10.1016/S0140-6736(03)13861-5

[50] Austin, P. C., Escobar, M., & Kopec, J. A. (2000). The use of the Tobit model for analyzing measures of health status. *Quality of Life Research*, *9*, 901–910. 11284209 10.1023/a:1008938326604

[51] Hunger, M., Döring, A., & Holle, R. (2012). Longitudinal beta regression models for analyzing health-related quality of life scores over time. *BMC Medical Research Methodology*, *12*, 1–12. 22984825 10.1186/1471-2288-12-144 PMC3528618

[52] Pullenayegum, E. M., Tarride, J. E., Xie, F., Goeree, R., Gerstein, H. C., & O’Reilly, D. (2010). Analysis of health utility data when some subjects attain the upper bound of 1: Are Tobit and CLAD models appropriate? *Value in Health*, *13*(4), 487–494. 20230549 10.1111/j.1524-4733.2010.00695.x

[53] Watkinson, R. E., Sutton, M., & Turner, A. J. (2021). Ethnic inequalities in health-related quality of life among older adults in England: Secondary analysis of a national cross-sectional survey. *The Lancet Public Health*, *6*(3), e145–e154. 33516278 10.1016/S2468-2667(20)30287-5

[54] Li, D-L., Wang, Z-T., Nie, X-Y., Luo, N., Wu, Y-B., Pan, C-W., & Wang, P. (2024). EQ-5D-5L population norms for China derived from a national health survey. *Value in Health*, *27*(8), 1108–1120. 38677363 10.1016/j.jval.2024.04.014

[55] Purba, F. D., Hunfeld, J. A. M., Fitriana, T. S., Iskandarsyah, A., Sadarjoen, S. S., Busschbach, J. J. V., & Passchier, J. (2018). Living in uncertainty due to floods and pollution: The health status and quality of life of people living on an unhealthy riverbank. *Bmc Public Health*, *18*(1), 782. 29929524 10.1186/s12889-018-5706-0 PMC6013864

[56] Kohler, S., Sidney Annerstedt, K., Diwan, V., Lindholm, L., Randive, B., Vora, K., & De Costa, A. (2018). Postpartum quality of life in Indian women after vaginal birth and cesarean section: A pilot study using the EQ-5D-5L descriptive system. *Bmc Pregnancy and Childbirth*, *18*(1), 427. 30373545 10.1186/s12884-018-2038-0 PMC6206933

[57] Chen, Y., Hicks, A., & While, A. E. (2013). Quality of life of older people in China: A systematic review. *Reviews in Clinical Gerontology*, *23*(1), 88–100.

[58] Farajzadeh, M., Gheshlagh, R. G., & Sayehmiri, K. (2017). Health related quality of life in Iranian elderly citizens: A systematic review and meta-analysis. *International Journal of Community Based Nursing and Midwifery*, *5*(2), 100. 28409164 PMC5385233

[59] Fahad Saleem, F. S., Mohamed Azmi Hassali, M. A. H., Asrul Akmal Shafie, A. A. S., Awad, G., Muhammad Atif, M. A., Noman-Ul-Haq N-u-H, Hisham Aljadhey, H. A., & Maryam Farooqui, M. F. (2012). Does treatment adherence correlates with health related quality of life? Findings from a cross sectional study. *BMC Public Health, 12*, 318. 10.1186/1471-2458-12-318 PMC3488479 22545950

[60] Prabowo, M. H., Febrinasari, R. P., Pamungkasari, E. P., Mahendradhata, Y., Pulkki-Brannstrom, A. M., & Probandari, A. (2023). Health-related quality of life of patients with Diabetes Mellitus measured with the Bahasa Indonesia Version of EQ-5D in primary care settings in Indonesia. *J Prev Med Public Health*, *56*(5), 467–474. 37828874 10.3961/jpmph.23.229 PMC10579634

[61] Buck, D., Jacoby, A., Baker, G. A., Ley, H., & Steen, N. (1999). Cross-cultural differences in health-related quality of life of people with epilepsy: Findings from a European study. *Quality of life Research*, *8*, 675–685. 10855341 10.1023/a:1008916326411

