# Supplementary material for: Socio-demographic disparities in health-related quality of life in hypertensive patients in Bangladesh: a comprehensive survey analysis
Source: Qual Life Res. 2025 Feb 6;34(5):1457–71. doi: 10.1007/s11136-025-03912-3 (PMC12064452; doi:10.1007/s11136-025-03912-3)
Supplement: Supplementary file 1 — Supplementary Material 1 [file 11136_2025_3912_MOESM1_ESM.docx]

**Appendix**

**Socio-demographic disparities in health-related quality of life in hypertensive patients in Bangladesh: a comprehensive survey analysis**

**Table S1: Study characteristics of eligible studies in South Asian countries (n=35 studies)**

| **Study** | **Survey year** | **Country** | **Sample size** | **Focus population** | **Name of quality index** | **EQ-5D index** | **VAS-index** |
| --- | --- | --- | --- | --- | --- | --- | --- |
| Polack et al, 2010[1] | 2005-2006 | Bangladesh | 1212 | Adults with visually impairing cataract | EQ-5D-3L | NA | NA |
| Sultana et al, 2016[2] | 2014 | Bangladesh | 1433 | Low-income communities | EQ-5D-3L, VAS-index | 0.76 | 77.00 |
| Mahumud et al, 2019[3] | 2015-2016 | Bangladesh | 465 | Pregnant women | EQ-5D-3L, VAS-index | 0.67 | 79.00 |
| Barua et al, 2021[4] | 2018 | Bangladesh | 1806 | Type 2 diabetes population | EQ-5D-5L | 0.62 | NA |
| Mannan et al, 2022[5] | 2020-2021 | Bangladesh | 1912 | Patients with hypertension | EQ-5D-3L | 0.64, 0.59, 0.58 | NA |
| Namdeo et al, 2023[6] | 2014-2015 | Bangladesh | 318 | Type 2 diabetes population | EQ-5D-5L, VAS-index | 0.62 | 69.00 |
| Singh et al, 2017[7] | 2010-2011 | India | 16287 | General population | EQ-5D-3L, VAS-index | NA | 74.50 |
| Kohler et al, 2018[8] | 2014-2016 | India | 224 | Postpartum women | EQ-5D-5L | 0.77, 0.66 | NA |
| Muhammed et al, 2018[9] | 2015-2016 | India | 101 | Patients with systemic lupus erythematosus | EQ-5D-3L, VAS-index | NA | 54.30 |
| Jyani et al, 2022[10] | 2019-2020 | India | 2409 | General population | EQ-5D-5L, VAS-index | 0.85 | 75.18 |
| Jyani et al, 2023[11] | 2019-2020 | India | 2307 | General population | EQ-5D-5L, VAS-index | 0.84 | 75.28 |
| Purba et al, 2017 (b)[12] | 2015-2016 | Indonesia | 1054 | General population | EQ-5D-5L, VAS-index | NA | 79.38 |
| Setyowibowo et al, 2018[13] | NA | Indonesia | 603 | Women with breast cancer symptoms | EQ-5D-5L, VAS-index | 0.80 | 69.10 |
| Purba et al, 2018 (a)[14] | 2015 | Indonesia | 1754 | Individuals living on Ciliwung River | EQ-5D-5L, VAS-index | 0.91 | 81.74 |
| Rensa et al, 2019[15] | 2017 | Indonesia | 325 | Older women with low socioeconomic status | EQ-5D-3L, VAS-index | 76.80 | 70.00 |
| Fitriana et al, 2022[16] | 2019 | Indonesia | 1294 | General population | EQ-5D-3L | NA | NA |
| Saleem et al, 2012[17] | 2010 | Pakistan | 385 | Hypertensive patients | EQ-5D-3L, VAS-index | 0.46 | 63.97 |
| Saleem et al, 2018[18] | 2015-2016 | Pakistan | 226 | Pulmonary tuberculosis patients who were drug-susceptible | EQ-5D-3L, VAS-index | 0.43, 0.68, 0.88 | 54.73, 63.47, 76.56 |
| Ahmed et al, 2021[19] | 2019 | Pakistan | 602 | HIV/AIDS and receiving antiretroviral therapy | EQ-5D-3L, VAS-index | 0.39 | 66.20 |
| Kularatna et al, 2014[20] | 2012-2013 | Sri Lanka | 719 | General population | EQ-5D-3L | 0.85 | NA |
| Kularatna et al, 2015[21] | 2012-2013 | Sri Lanka | 736 | General population | EQ-5D-3L | NA | NA |
| Damayanthi et al, 2018[22] | 2015-2016 | Sri Lanka | 1300 | Older people | EQ-5D-3L | NA | NA |
| Kularatna et al, 2019[23] | 2015 | Sri Lanka | 1162 | Patients with chronic kidney disease | EQ-5D-3L | 0.54 | NA |
| Senanayake et al, 2019[24] | NA | Sri Lanka | 1036 | Patients with chronic kidney disease | EQ-5D-3L, VAS-index | 0.52 | 51.35 |
| Jayamaha et al, 2023[25] | NA | Sri Lanka | 464 | Patients with substance use disorders | EQ-5D-5L, VAS-index | 0.55, 0.81 | 50, 90 |
| Sakthong et al, 2015[26] | 2014-2015 | Thailand | 1156 | Patients with various chronic diseases | EQ-5D-5L, VAS-index | 0.86 | 78.00 |
| Muennig et al, 2015[27] | 2013 | Thailand | 102 | Adult refugees | EQ-5D-5L | 0.70 | NA |
| Sakthong and Munpan, 2017[28] | 2014-2015 | Thailand | 356 | Patients with chronic diseases | EQ-5D-5L, VAS-index | NA | NA |
| Pattanaphesaj et al, 2018[29] | 2013-2014 | Thailand | 1207 | General population | EQ-5D-5L, VAS-index | NA | 83.08 |
| Janssen et al, 2019[30] | 2007 | Thailand | 1409 | General population | EQ-5D-3L, VAS-index | NA | 78.90 |
| Kangwanrattanakul and Parmontree, 2020[31] | NA | Thailand | 1200 | General population | EQ-5D-3L, EQ-5D-5L, VAS-index | NA | NA |
| Taburee et al, 2020[32] | 2016 | Thailand | 384 | Older adults | EQ-5D-3L | 0.80 | NA |
| Aung et al, 2022[33] | 2019 | Thailand | 1509 | Older adults | EQ-5D-5L, VAS-index | 0.82 | 77.03 |
| Kaikeaw et al, 2023[34] | 2003, 2006, 2015, 2019 | Thailand | 12450, 12535, 29670, 16324 | General population | EQ-5D-5L | 0.832, 0.867, 0.934, 0.941 | NA |
| Kangwanrattanakul and Krägeloh, 2024[35] | 2023 | Thailand | 2000 | General population | EQ-5D-3L, EQ-5D-5L, VAS-index | 0.845, 0.923 | 79.83 |

**Table S2**: Population with various levels of problems across EQ-5D-5L dimensions in Bangladesh

| **EQ-5D-5L dimensions** | **Frequency (%)** | | | **p-value**  **(χ^2^-statistic)** |
| --- | --- | --- | --- | --- |
|  | **Female**  **(n=2887)** | **Male**  **(n=2199)** | **Both sexes**  **(n=5086)** |  |
| **Mobility** |  |  |  | <0.001 |
| No problems | 1475 (51.1) | 1400 (63.7) | 2875 (56.5) |  |
| Slight problems | 1122 (38.9) | 648 (29.5) | 1770 (34.8) |  |
| Moderate problems | 269 (9.3) | 132 (6.0) | 401 (7.9) |  |
| Severe problems | 20 (0.7) | 18 (0.8) | 38 (0.8) |  |
| Unable to | 1 (0.0) | 1 (0.1) | 2 (0.0) |  |
| **Self-care** |  |  |  | <0.001 |
| No problems | 1638 (56.7) | 1466 (66.7) | 3104 (61.0) |  |
| Slight problems | 988 (34.2) | 567 (25.8) | 1555 (30.6) |  |
| Moderate problems | 232 (8.0) | 140 (6.4) | 372 (7.3) |  |
| Severe problems | 28 (1.0) | 24 (1.1) | 52 (1.0) |  |
| Unable to | 1 (0.0) | 2 (0.1) | 3 (0.1) |  |
| **Activity** |  |  |  | <0.001 |
| No problems | 1455 (50.4) | 1343 (61.1) | 2798 (55.0) |  |
| Slight problems | 1199 (41.5) | 658 (29.9) | 1857 (36.5) |  |
| Moderate problems | 205 (7.1) | 163 (7.4) | 368 (7.2) |  |
| Severe problems | 26 (0.9) | 32 (1.5) | 58 (1.1) |  |
| Unable to | 2 (0.1) | 3 (0.1) | 5 (0.1) |  |
| **Pain** |  |  |  | <0.001 |
| No problems | 391 (13.5) | 594 (27.0) | 985 (19.4) |  |
| Slight problems | 1846 (63.9) | 1170 (53.2) | 3016 (59.3) |  |
| Moderate problems | 612 (21.2) | 412 (18.7) | 1024 (20.1) |  |
| Severe problems | 34 (1.2) | 22 (1.0) | 56 (1.1) |  |
| Unable to | 4 (0.1) | 1 (0.1) | 5 (0.1) |  |
| **Anxiety** |  |  |  | >0.100 |
| No problems | 539 (18.7) | 415 (18.9) | 954 (18.8) |  |
| Slight problems | 1860 (64.4) | 1376 (62.6) | 3236 (63.6) |  |
| Moderate problems | 455 (15.8) | 381 (17.3) | 836 (16.4) |  |
| Severe problems | 31 (1.1) | 24 (1.1) | 55 (1.1) |  |
| Unable to | 2 (0.1) | 3 (0.1) | 5 (0.1) |  |

**Table S3**: Problems in EQ-5D-5L dimensions by age groups for females in Bangladesh

| **EQ-5D-5L dimensions** | **Age groups (years) (%)** | | | | | | **p-value**  **(χ^2^-statistic)** |
| --- | --- | --- | --- | --- | --- | --- | --- |
|  | **≤30**  **n=156** | **31-39**  **n=288** | **40-49**  **n=780** | **50-59**  **n=778** | **60-69**  **n=627** | **≥70**  **n=258** |  |
| **Mobility** |  |  |  |  |  |  | <0.001 |
| No problems | 83.3 | 68.8 | 57.7 | 47.0 | 42.1 | 26.0 |  |
| Slight problems | 15.4 | 28.8 | 37.3 | 43.7 | 41.3 | 48.5 |  |
| Moderate problems | 1.3 | 2.4 | 4.7 | 8.5 | 15.3 | 23.6 |  |
| Severe problems | 0.0 | 0.0 | 0.3 | 0.8 | 1.1 | 1.9 |  |
| Unable to | 0.0 | 0.0 | 0.0 | 0.0 | 0.2 | 0.0 |  |
| **Self-care** |  |  |  |  |  |  | <0.001 |
| No problems | 84.6 | 72.2 | 64.6 | 54.2 | 47.4 | 29.1 |  |
| Slight problems | 12.8 | 25.4 | 31.4 | 36.3 | 38.6 | 48.8 |  |
| Moderate problems | 1.9 | 2.4 | 4.0 | 8.4 | 12.3 | 19.0 |  |
| Severe problems | 0.6 | 0.0 | 0.0 | 1.2 | 1.6 | 3.1 |  |
| Unable to | 0.0 | 0.0 | 0.0 | 0.0 | 0.2 | 0.0 |  |
| **Activity** |  |  |  |  |  |  | <0.001 |
| No problems | 82.1 | 69.8 | 54.9 | 46.9 | 42.0 | 27.1 |  |
| Slight problems | 16.7 | 26.7 | 41.0 | 44.1 | 46.7 | 54.3 |  |
| Moderate problems | 1.3 | 3.1 | 4.0 | 8.1 | 9.1 | 16.7 |  |
| Severe problems | 0.0 | 0.4 | 0.1 | 0.8 | 2.1 | 1.9 |  |
| Unable to | 0.0 | 0.0 | 0.0 | 0.1 | 0.2 | 0.0 |  |
| **Pain** |  |  |  |  |  |  | <0.001 |
| No problems | 41.7 | 22.9 | 15.1 | 10.4 | 6.7 | 7.4 |  |
| Slight problems | 50.6 | 62.9 | 65.6 | 65.0 | 66.5 | 58.5 |  |
| Moderate problems | 7.7 | 13.5 | 18.5 | 23.3 | 24.2 | 32.6 |  |
| Severe problems | 0.0 | 0.7 | 0.3 | 1.3 | 2.6 | 1.6 |  |
| Unable to | 0.0 | 0.0 | 0.5 | 0.0 | 0.0 | 0.0 |  |
| **Anxiety** |  |  |  |  |  |  | <0.001 |
| No problems | 36.5 | 24.3 | 18.1 | 15.6 | 16.0 | 19.4 |  |
| Slight problems | 58.3 | 62.9 | 66.4 | 64.4 | 65.1 | 62.4 |  |
| Moderate problems | 5.1 | 12.2 | 14.6 | 18.9 | 17.5 | 15.9 |  |
| Severe problems | 0.0 | 0.7 | 0.9 | 1.0 | 1.4 | 1.9 |  |
| Unable to | 0.0 | 0.0 | 0.0 | 0.1 | 0.0 | 0.4 |  |

**Table S4:** Problems in EQ-5D-5L dimensions by age groups for males in Bangladesh

| **EQ-5D-5L dimensions** | **Age groups (years) (%)** | | | | | | **p-value**  **(χ^2^-statistic)** |
| --- | --- | --- | --- | --- | --- | --- | --- |
|  | **≤30**  **n=** **98** | **31-39**  **n=** **254** | **40-49**  **n=** **404** | **50-59**  **n=** **620** | **60-69**  **n=** **562** | **≥70**  **n=** **261** |  |
| **Mobility** |  |  |  |  |  |  | <0.001 |
| No problems | 90.8 | 90.2 | 79.5 | 63.4 | 49.5 | 34.5 |  |
| Slight problems | 8.2 | 7.9 | 17.8 | 32.4 | 39.7 | 47.5 |  |
| Moderate problems | 0.0 | 1.6 | 2.7 | 3.7 | 9.1 | 16.5 |  |
| Severe problems | 1.0 | 0.4 | 0.0 | 0.5 | 1.6 | 1.5 |  |
| Unable to | 0.0 | 0.0 | 0.0 | 0.0 | 0.2 | 0.0 |  |
| **Self-care** |  |  |  |  |  |  | <0.001 |
| No problems | 92.9 | 91.7 | 81.7 | 64.0 | 55.5 | 39.5 |  |
| Slight problems | 6.1 | 6.7 | 15.1 | 30.0 | 33.1 | 42.5 |  |
| Moderate problems | 0.0 | 1.2 | 3.2 | 5.3 | 9.1 | 15.3 |  |
| Severe problems | 1.0 | 0.4 | 0.0 | 0.5 | 2.1 | 2.7 |  |
| Unable to | 0.0 | 0.0 | 0.0 | 0.2 | 0.2 | 0.0 |  |
| **Activity** |  |  |  |  |  |  | <0.001 |
| No problems | 88.8 | 88.2 | 74.5 | 58.7 | 48.8 | 35.6 |  |
| Slight problems | 9.2 | 10.2 | 21.0 | 31.6 | 39.3 | 46.4 |  |
| Moderate problems | 1.0 | 1.2 | 4.0 | 8.6 | 8.9 | 15.3 |  |
| Severe problems | 0.0 | 0.4 | 0.5 | 1.0 | 2.9 | 2.7 |  |
| Unable to | 1.0 | 0.0 | 0.0 | 0.2 | 0.2 | 0.0 |  |
| **Pain** |  |  |  |  |  |  | <0.001 |
| No problems | 67.4 | 49.2 | 35.2 | 23.6 | 15.0 | 11.9 |  |
| Slight problems | 29.6 | 44.5 | 49.0 | 55.2 | 58.5 | 60.9 |  |
| Moderate problems | 2.0 | 5.9 | 15.6 | 20.2 | 24.9 | 25.7 |  |
| Severe problems | 1.0 | 0.4 | 0.3 | 1.0 | 1.6 | 1.5 |  |
| Unable to | 0.0 | 0.0 | 0.0 | 0.2 | 0.0 | 0.0 |  |
| **Anxiety** |  |  |  |  |  |  | <0.01 |
| No problems | 28.6 | 23.6 | 19.8 | 18.4 | 14.8 | 19.2 |  |
| Slight problems | 61.2 | 65.8 | 62.9 | 61.9 | 62.5 | 61.3 |  |
| Moderate problems | 9.2 | 9.8 | 15.8 | 19.0 | 20.8 | 18.4 |  |
| Severe problems | 1.0 | 0.8 | 1.5 | 0.7 | 1.6 | 0.8 |  |
| Unable to | 0.0 | 0.0 | 0.0 | 0.0 | 0.4 | 0.4 |  |

**Table S5: Logit-transformed r**egression analyses on health-related quality of life scores, Bangladesh

|  | **EQ-5D index** | |  | **EQ-VAS score** | |
| --- | --- | --- | --- | --- | --- |
|  | \| Coefficient \| \| --- \| | **p-values** |  | Coefficient | **p-values** |
| **Age, years** |  |  |  |  |  |
| ≤30 | 0 |  |  | 0 |  |
| 31-39 | -0.356 (-0.528–-0.183) | **<0.01** |  | -0.228 (-0.341–-0.116) | **<0.01** |
| 40-49 | -0.689 (-0.85–-0.529) | **<0.01** |  | -0.472 (-0.577–-0.367) | **<0.01** |
| 50-59 | -0.938 (-1.099–-0.776) | **<0.01** |  | -0.595 (-0.702–-0.489) | **<0.01** |
| 60-69 | -1.059 (-1.227–-0.891) | **<0.01** |  | -0.748 (-0.859–-0.637) | **<0.01** |
| ≥70 | -1.329 (-1.516–-1.143) | **<0.01** |  | -0.806 (-0.93–-0.683) | **<0.01** |
| **Gender** |  |  |  |  |  |
| Female | 0 |  |  | 0 |  |
| Male | 0.195 (0.018–0.372) | 0.031 |  | 0.191 (0.072–0.309) | 0.002 |
| **Education** |  |  |  |  |  |
| No education | 0 |  |  | 0 |  |
| Primary | 0.075 (-0.004–0.155) | 0.063 |  | 0.081 (0.027–0.135) | 0.003 |
| Secondary | 0.262 (0.164–0.36) | **<0.01** |  | 0.182 (0.116–0.249) | **<0.01** |
| Higher | 0.311 (0.197–0.424) | **<0.01** |  | 0.274 (0.198–0.35) | **<0.01** |
| **Religion** |  |  |  |  |  |
| Muslim | 0 |  |  | 0 |  |
| Non-Muslim | -0.117 (-0.259–0.026) | 0.108 |  | 0.018 (-0.078–0.115) | 0.708 |
| **Marital status** |  |  |  |  |  |
| Married | 0 |  |  | 0 |  |
| Others | -0.033 (-0.125–0.06) | 0.489 |  | -0.094 (-0.157–-0.031) | 0.004 |
| **Household size** |  |  |  |  |  |
| 1–2 | 0 |  |  | 0 |  |
| 3–4 | -0.124 (-0.219–-0.029) | 0.010 |  | -0.021 (-0.086–0.043) | 0.515 |
| ≥5 | -0.103 (-0.196–-0.011) | 0.029 |  | 0.033 (-0.031–0.096) | 0.311 |
| **Occupation** |  |  |  |  |  |
| Not working | 0 |  |  | 0 |  |
| Working | 0.151 (-0.028–0.331) | 0.098 |  | 0.02 (-0.1–0.141) | 0.740 |
| Retired | -0.523 (-0.743–-0.304) | **<0.01** |  | -0.376 (-0.523–-0.229) | 0.000 |
| Others | 0.297 (-0.024–0.617) | 0.069 |  | 0.137 (-0.079–0.353) | 0.213 |
| **BMI status** |  |  |  |  |  |
| Poor | 0 |  |  | 0 |  |
| Intermediate | -0.026 (-0.129–0.077) | 0.619 |  | 0.031 (-0.038–0.1) | 0.383 |
| Ideal | -0.024 (-0.123–0.075) | 0.637 |  | 0.041 (-0.025–0.108) | 0.223 |
| **Blood pressure status** |  |  |  |  |  |
| Poor | 0 |  |  | 0 |  |
| Intermediate | -0.012 (-0.081–0.057) | 0.729 |  | 0.028 (-0.019–0.074) | 0.244 |
| Ideal | 0.037 (-0.074–0.147) | 0.518 |  | 0.069 (-0.005–0.144) | 0.068 |
| **FBG** |  |  |  |  |  |
| Poor | 0 |  |  | 0 |  |
| Intermediate | 0 (-0.067–0.067) | 0.996 |  | -0.011 (-0.056–0.034) | 0.642 |
| Ideal | 0.045 (-0.039–0.129) | 0.291 |  | -0.036 (-0.092–0.021) | 0.215 |
| **Place of residence** |  |  |  |  |  |
| Urban | 0 |  |  | 0 |  |
| Rural | 0.04 (-0.039–0.12) | 0.319 |  | 0.056 (0.003–0.11) | 0.037 |
| **Expenditure quintile** |  |  |  |  |  |
| Quint1 (poorest) | 0 |  |  | 0 |  |
| Quint2 | 0.126 (0.032–0.22) | 0.009 |  | 0.043 (-0.021–0.106) | 0.190 |
| Quint3 | 0.111 (0.012–0.209) | 0.028 |  | 0.041 (-0.026–0.107) | 0.229 |
| Quint4 | 0.123 (0.022–0.223) | 0.017 |  | -0.03 (-0.098–0.039) | 0.395 |
| Quint5 (richest) | -0.054 (-0.167–0.059) | 0.351 |  | -0.14 (-0.216–-0.064) | **<0.01** |

Others refers to single/Divorced/widowed/Separated); BMI, body mass index; FBG, fasting blood glucose

**Blood pressure**: Poor: When SBP ≥140 or DBP≥90 mm Hg; Intermediate: When SBP 120–139 or DBP 80–89 or treated BP <140/<90 mm Hg; Ideal: When BP <120 and <80 mm Hg.

**BMI:** Poor: When BMI is 30 or greater; Intermediate: When BMI is between 25 and 29.9; Ideal: When BMI is less than 25;

**FBG:** Poor: When FBG is 126 mg/dL or greater; Intermediate: When FBG is between 100 and 125 mg/dL; Ideal: When FBG is less than 100 mg/dL


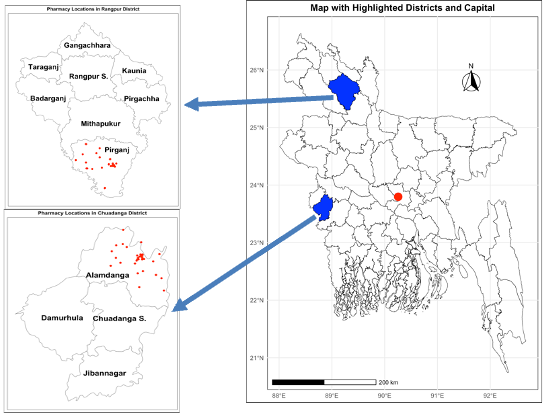


**Figure S1**: Study area selection, Bangladesh

| 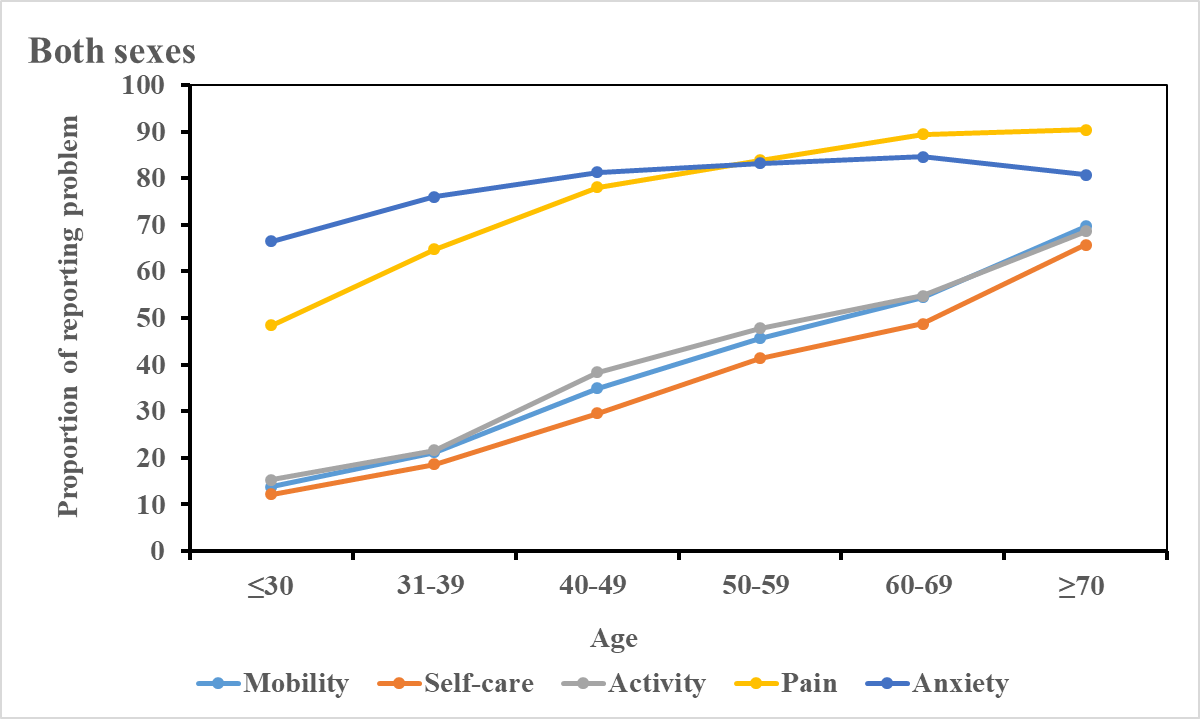 |
| --- |
| 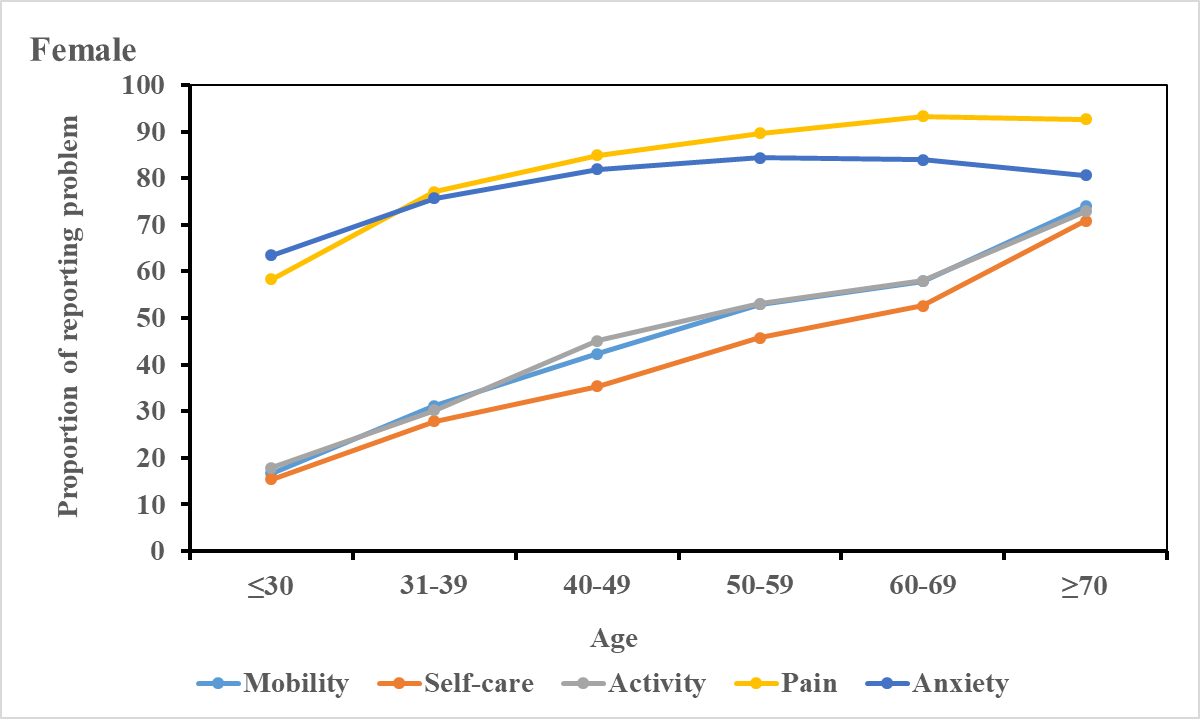 |
| 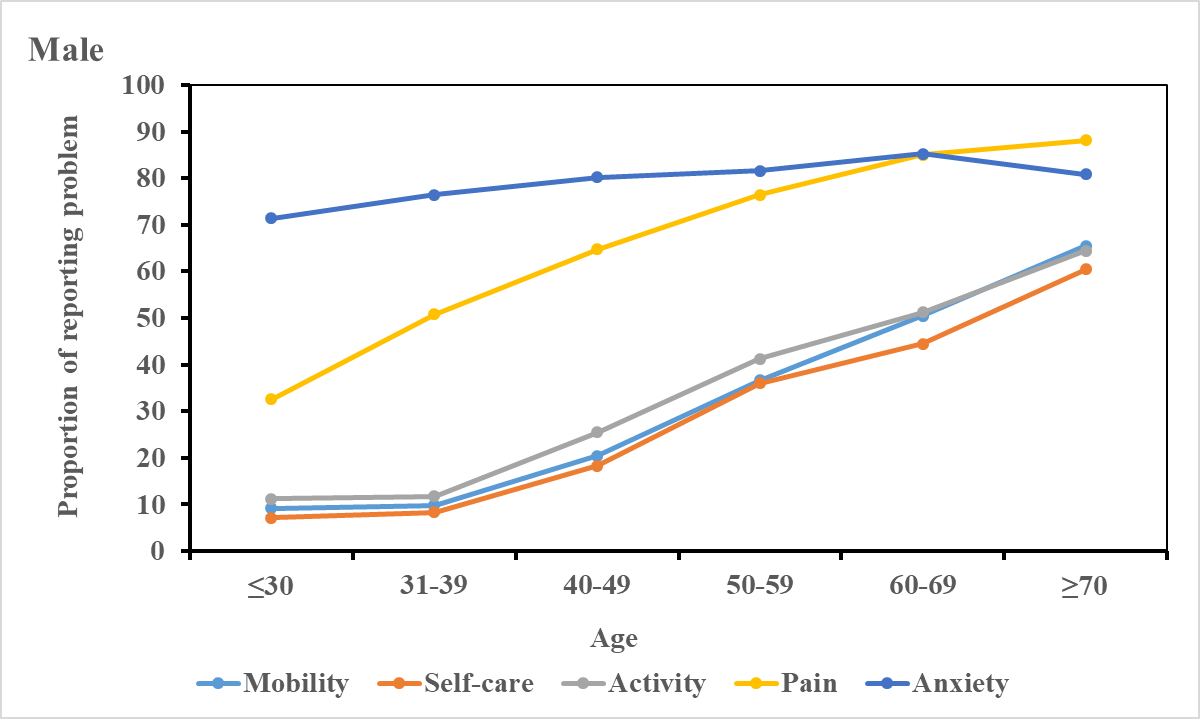 |

**Figure S2**: Percentage of reporting any problems in 5 health domains

**Rerferences**

1. Polack, S., Eusebio, C., Mathenge, W., Wadud, Z., Mamunur, A. K., Fletcher, A., Foster, A., & Kuper, H. (2010). The impact of cataract surgery on health related quality of life in Kenya, the Philippines, and Bangladesh. Ophthalmic Epidemiol, 17(6), 387-399.

2. Sultana, M., Sarker, A. R., Mahumud, R. A., Ahmed, S., Ahmed, W., Chakrovorty, S., Rahman, H., Islam, Z., & Khan, J. A. (2016). Inequalities in Health Status from EQ-5D Findings: A Cross-Sectional Study in Low-Income Communities of Bangladesh. Int J Health Policy Manag, 5(5), 301-308.

3. Mahumud, R. A., Ali, N., Sheikh, N., Akram, R., Alam, K., Gow, J., Sarker, A. R., & Sultana, M. (2019). Measuring perinatal and postpartum quality of life of women and associated factors in semi-urban Bangladesh. Qual Life Res, 28(11), 2989-3004.

4. Barua, L., Faruque, M., Chowdhury, H. A., Banik, P. C., & Ali, L. (2021). Health-related quality of life and its predictors among the type 2 diabetes population of Bangladesh: A nation-wide cross-sectional study. J Diabetes Investig, 12(2), 277-285.

5. Mannan, A., Akter, K. M., Akter, F., Chy, N. U. H. A., Alam, N., Pinky, S. D., Chowdhury, A. F. M. N., Biswas, P., Chowdhury, A. S., & Hossain, M. A. (2022). Association between comorbidity and health-related quality of life in a hypertensive population: a hospital-based study in Bangladesh. BMC Public Health, 22(1), 181.

6. Namdeo, M. K., Verma, S., Das Gupta, R., Islam, R., Nazneen, S., & Rawal, L. B. (2023). Depression and health-related quality of life of patients with type 2 diabetes attending tertiary level hospitals in Dhaka, Bangladesh. Glob Health Res Policy, 8(1), 43.

7. Singh, K., Kondal, D., Shivashankar, R., Ali, M. K., Pradeepa, R., Ajay, V. S., Mohan, V., Kadir, M. M., Sullivan, M. D., Tandon, N., Narayan, K. M. V., & Prabhakaran, D. (2017). Health-related quality of life variations by sociodemographic factors and chronic conditions in three metropolitan cities of South Asia: the CARRS study. BMJ Open, 7(10), e018424.

8. Kohler, S., Sidney Annerstedt, K., Diwan, V., Lindholm, L., Randive, B., Vora, K., & De Costa, A. (2018). Postpartum quality of life in Indian women after vaginal birth and cesarean section: a pilot study using the EQ-5D-5L descriptive system. BMC Pregnancy Childbirth, 18(1), 427.

9. Muhammed, H., Goyal, M., Lal, V., Singh, S., & Dhir, V. (2018). Neuropsychiatric manifestations are not uncommon in Indian lupus patients and negatively affect quality of life. Lupus, 27(4), 688-693.

10. Jyani, G., Sharma, A., Prinja, S., Kar, S. S., Trivedi, M., Patro, B. K., Goyal, A., Purba, F. D., Finch, A. P., Rajsekar, K., Raman, S., Stolk, E., & Kaur, M. (2022). Development of an EQ-5D Value Set for India Using an Extended Design (DEVINE) Study: The Indian 5-Level Version EQ-5D Value Set. Value Health, 25(7), 1218-1226.

11. Jyani, G., Prinja, S., Garg, B., Kaur, M., Grover, S., Sharma, A., & Goyal, A. (2023). Health-related quality of life among Indian population: The EQ-5D population norms for India. J Glob Health, 13, 04018.

12. Purba, F. D., Hunfeld, J. A. M., Iskandarsyah, A., Fitriana, T. S., Sadarjoen, S. S., Ramos-Goni, J. M., Passchier, J., & Busschbach, J. J. V. (2017). The Indonesian EQ-5D-5L Value Set. Pharmacoeconomics, 35(11), 1153-1165.

13. Setyowibowo, H., Purba, F. D., Hunfeld, J. A. M., Iskandarsyah, A., Sadarjoen, S. S., Passchier, J., & Sijbrandij, M. (2018). Quality of life and health status of Indonesian women with breast cancer symptoms before the definitive diagnosis: A comparison with Indonesian women in general. PLoS One, 13(7), e0200966.

14. Purba, F. D., Hunfeld, J. A. M., Fitriana, T. S., Iskandarsyah, A., Sadarjoen, S. S., Busschbach, J. J. V., & Passchier, J. (2018). Living in uncertainty due to floods and pollution: the health status and quality of life of people living on an unhealthy riverbank. BMC Public Health, 18(1), 782.

15. Rensa, R., Setiati, S., Laksmi, P. W., & Rinaldi, I. (2019). Factors associated with physical frailty in elderly women with low socioeconomic status in urban communities: a cross-sectional study. Acta Med Indones, 51(3), 220-229.

16. Fitriana, T. S., Roudijk, B., Purba, F. D., Busschbach, J. J. V., & Stolk, E. (2022). Estimating an EQ-5D-Y-3L Value Set for Indonesia by Mapping the DCE onto TTO Values. Pharmacoeconomics, 40(Suppl 2), 157-167.

17. Fahad Saleem, F. S., Mohamed Azmi Hassali, M. A. H., Asrul Akmal Shafie, A. A. S., Awad, G., Muhammad Atif, M. A., Noman-ul-Haq, N.-u.-H., Hisham Aljadhey, H. A., & Maryam Farooqui, M. F. (2012). Does treatment adherence correlates with health related quality of life? Findings from a cross sectional study.

18. Saleem, S., A, A. M., Ghulam, A., Ahmed, J., & Hussain, H. (2018). Health-related quality of life among pulmonary tuberculosis patients in Pakistan. Qual Life Res, 27(12), 3137-3143.

19. Ahmed, A., Saqlain, M., Bashir, N., Dujaili, J., Hashmi, F., Mazhar, F., Khan, A., Jabeen, M., Blebil, A., & Awaisu, A. (2021). Health-related quality of life and its predictors among adults living with HIV/AIDS and receiving antiretroviral therapy in Pakistan. Qual Life Res, 30(6), 1653-1664.

20. Kularatna, S., Whitty, J. A., Johnson, N. W., Jayasinghe, R., & Scuffham, P. A. (2014). EQ-5D-3L derived population norms for health related quality of life in Sri Lanka. PLoS One, 9(11), e108434.

21. Kularatna, S., Whitty, J. A., Johnson, N. W., Jayasinghe, R., & Scuffham, P. A. (2015). Valuing EQ-5D health states for Sri Lanka. Qual Life Res, 24(7), 1785-1793.

22. Damayanthi, H., Moy, F. M., Abdullah, K. L., & Dharmaratne, S. D. (2018). Health related quality of life and its associated factors among community-dwelling older people in Sri Lanka: A cross-sectional study. Arch Gerontol Geriatr, 76, 215-220.

23. Kularatna, S., Senanayake, S., Gunawardena, N., & Graves, N. (2019). Comparison of the EQ-5D 3L and the SF-6D (SF-36) contemporaneous utility scores in patients with chronic kidney disease in Sri Lanka: a cross-sectional survey. BMJ Open, 9(2), e024854.

24. Senanayake, S., Mahesh, P. K. B., Gunawardena, N., Graves, N., & Kularatna, S. (2019). Validity and internal consistency of EQ-5D-3L quality of life tool among pre-dialysis patients with chronic kidney disease in Sri Lanka, a lower middle-income country. PLoS One, 14(6), e0211604.

25. Jayamaha, A. R., Herath, N. D. M., Dharmarathna, N. D., Sandakumari, H. S., Ranadeva, N. D. K., Fernando, M. M., Samarakoon, N. A. W., Amarabandu, P. N., Senanayake, B., Darshana, T., Renuka, N., Samarasinghe, K. L., & Fernando, N. (2023). Health-related quality of life in patients with substance use disorders enrolled to the residential treatment in Sri Lanka: a retrospective cross-sectional study. Qual Life Res, 32(2), 435-445.

26. Sakthong, P., Sonsa-Ardjit, N., Sukarnjanaset, P., & Munpan, W. (2015). Psychometric properties of the EQ-5D-5L in Thai patients with chronic diseases. Qual Life Res, 24(12), 3015-3022.

27. Muennig, P., Boulmier-Darden, P., Khouzam, N., Zhu, W., & Hancock, P. (2015). Predictors of Health Among Refugee Adults from Myanmar and the Development of Their Children. J Immigr Minor Health, 17(5), 1385-1390.

28. Sakthong, P., & Munpan, W. (2017). A Head-to-Head Comparison of UK SF-6D and Thai and UK EQ-5D-5L Value Sets in Thai Patients with Chronic Diseases. Appl Health Econ Health Policy, 15(5), 669-679.

29. Pattanaphesaj, J., Thavorncharoensap, M., Ramos-Goni, J. M., Tongsiri, S., Ingsrisawang, L., & Teerawattananon, Y. (2018). The EQ-5D-5L Valuation study in Thailand. Expert Rev Pharmacoecon Outcomes Res, 18(5), 551-558.

30. Janssen, M. F., Szende, A., Cabases, J., Ramos-Goni, J. M., Vilagut, G., & Konig, H. H. (2019). Population norms for the EQ-5D-3L: a cross-country analysis of population surveys for 20 countries. Eur J Health Econ, 20(2), 205-216.

31. Kangwanrattanakul, K., & Parmontree, P. (2020). Psychometric properties comparison between EQ-5D-5L and EQ-5D-3L in the general Thai population. Qual Life Res, 29(12), 3407-3417.

32. Taburee, W., Sirilak, S., Khotcharrat, R., Anekpunyakul, P., Dilokthornsakul, P., Lukkahatai, N., & Boongird, C. (2020). Health-Related Problems and Drivers of Health-Related Quality of Life Among Community-Dwelling Older Adults. J Prim Care Community Health, 11, 2150132720913724.

33. Aung, T. N. N., Moolphate, S., Koyanagi, Y., Angkurawaranon, C., Supakankunti, S., Yuasa, M., & Aung, M. N. (2022). Determinants of Health-Related Quality of Life Among Community-Dwelling Thai Older Adults in Chiang Mai, Northern Thailand. Risk Manag Healthc Policy, 15, 1761-1774.

34. Kaikeaw, S., Punpuing, S., Chamchan, C., & Prasartkul, P. (2023). Socioeconomic inequalities in health outcomes among Thai older population in the era of Universal Health Coverage: trends and decomposition analysis. Int J Equity Health, 22(1), 144.

35. Kangwanrattanakul, K., & Krageloh, C. U. (2024). EQ-5D-3L and EQ-5D-5L population norms for Thailand. BMC Public Health, 24(1), 1108.
